# Do Wolves Protect Forests? Investigating the Link Between Wolf Density, Deer Browse, and Plant Recovery

**DOI:** 10.1002/ece3.72266

**Published:** 2025-10-16

**Authors:** Elaine M. Brice, Jennifer Price Tack, Amanda M. McGraw, Bernd Blossey

**Affiliations:** ^1^ Department of Natural Resources and the Environment Cornell University Ithaca New York USA; ^2^ Department of Biological Sciences Wellesley College Wellesley Massachusetts USA; ^3^ Wisconsin Department of Natural Resources Rhinelander Wisconsin USA; ^4^ Minnesota Department of Natural Resources Grand Rapids Minnesota USA

**Keywords:** deer browsing, forest regeneration, trophic cascade, white‐tailed deer, wolves

## Abstract

Large ungulate populations can threaten forest regeneration and many rare or declining understory plants, birds, and small mammals. Reintroduction of large predators is often proposed as a remedy to reduce negative ecosystem effects associated with high ungulate populations, but we know little about the effectiveness of this approach. We assessed whether wolves ( *Canis lupus* ) can protect forest understory plants from excessive white‐tailed deer ( *Odocoileus virginianus* ) browse. We planted white oak ( *Quercus alba* ), red oak ( *Quercus rubra* ), and zigzag goldenrod ( *Solidago flexicaulis* ) seedlings across a gradient of wolf density and residence time in northern Wisconsin and recorded deer browse and frequency of 11 common understory plants at each site. We found that wolf density and residence time had negative effects on deer browse intensity, but these effects were generally weak except when understory vegetation was abundant. Additionally, the presence of common understory plants decreased as a function of wolf density, opposite to what we would expect for a wolf‐driven trophic cascade. The weak reduction in browse probability that is associated with wolves, particularly when vegetation is scarce, is unlikely to improve forest regeneration and recovery of understory plant communities currently threatened by high deer populations.

## Graphical abstract

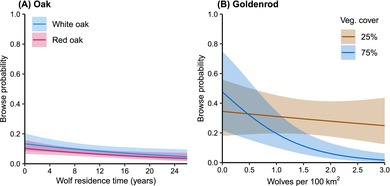

## 1 Introduction

Around the world, native ungulates are keystone species in forest ecosystems, regulating forest structure, composition, and biodiversity (Bernes et al. 2018). Over the last century, removal of top predators, increased subsidies from agriculture, forestry, and landscaping, stricter hunting regulations, and less severe winters associated with climate change have facilitated unprecedented ungulate population increases and impacts (Rooney 2001; Côté et al. 2004; Bernes et al. 2018; Ramirez et al. 2018). The challenge of managing forests and the surrounding landscapes experiencing high‐density ungulate populations (i.e., populations with browse rates exceeding long‐term viability and sustainability of the native vegetation) is exemplified by eastern temperate northern hardwood forests found along the border of the United States and Canada. Here, browsing by white‐tailed deer ( *Odocoileus virginianus* , hereafter “deer”) has been a major challenge for biodiversity conservation since at least the 1940s (Leopold et al. 1947; Blossey et al. 2024).

Deer are selective foragers, consuming diverse but high‐quality diets that vary seasonally and regionally (McCaffery et al. 1974; Rogers et al. 1981; Daigle et al. 2004; Côté et al. 2004; Frerker et al. 2013; Bradshaw and Waller 2016; Berry et al. 2019). Generally, deer diets are largely herbaceous (~87%) during the growing season, but also contain fruits/flowers (~10%) and woody browse (3%, McCaffery et al. 1974), and can be comprised of > 60% woody browse in winter because of a lack of other available forage in heavily forested regions (Kohn and Mooty 1971). The most well‐known ecological impact of high deer abundance is on forest regeneration by preventing seedling recruitment into the overstory, creating a widespread regeneration debt (Patton et al. 2018; Miller and McGill 2019; Miller et al. 2023). However, deer browse impacts extend beyond those on forest regeneration. The wide‐ranging negative consequences of reducing survival, growth, and regeneration of many woody and herbaceous plants (Jenkins et al. 2015; Pendergast et al. 2016; Bradshaw and Waller 2016; Blossey et al. 2019) can affect the abundance and diversity of insects (Chips et al. 2015) and birds (Rushing et al. 2020), alter nutrient and carbon cycling (Wardle et al. 2001; Côté et al. 2004), and facilitate the spread of tick‐borne diseases (Telford 2017) and invasive species (Averill et al. 2016, 2018), including non‐native earthworms (Dávalos, Simpson, et al. 2015).

State wildlife agencies charged with managing deer populations and their impacts mostly rely on recreational hunting and special shooting permits for forestry and agricultural damage to reduce deer abundance locally. However, these efforts have failed to stop or reverse landscape‐level impacts of deer on forest regeneration and biodiversity (Porter and Underwood 1999; Blossey et al. 2019, 2024; Nagy et al. 2022). Declines in the number of hunters (U.S. Fish and Wildlife Service and U.S. Census Bureau 2016), coupled with the fact that hunters are largely unwilling to harvest more deer than they can consume (1.8 deer in Wisconsin; Bradshaw et al. 2021), make traditional recreational harvest an ineffective tool for reducing deer densities. Alternative approaches, such as sterilization, are neither approved nor available at large spatial scales and appear to offer no promise of relief for ecological recovery when implemented experimentally (Blossey et al. 2019).

The failure of current deer management approaches to achieve conservation goals has led to proposals to consider large predator reintroductions (Wilson 2004; Nilsen et al. 2007; Allen et al. 2017; Wolf and Ripple 2018; Kirkland et al. 2021). Wolves ( *Canis lupus* ) and cougars ( *Puma concolor* ), although previously widespread, are now largely absent throughout much of the USA's northern hardwood forests. Proponents of predator reintroductions anticipate that predators will reduce ungulate densities through consumption or by changing foraging behavior, and thereby reduce the negative effects of ungulate herbivory on plants and abiotic functions (Ripple et al. 2014), a process known as a trophic cascade (Paine 1980; Estes et al. 2011). For example, the decline of elk ( *Cervus elaphus* ) populations following wolf reintroductions to Yellowstone National Park and the recovery of aspen ( *Populus tremuloides* ) and willow (*Salix* spp.) is frequently cited as the textbook case of a trophic cascade (Estes et al. 2011; Ripple et al. 2015; Painter et al. 2018). However, the strength of a wolf‐driven trophic cascade is considerably weaker than previously advocated and required additional elk predators, including cougars, bears, and humans, to decrease elk abundance enough to allow for an increase in plant growth (Kauffman et al. 2010; Winnie 2012; Fleming 2019; Brice et al. 2022, 2024; Hobbs et al. 2024).

Evidence for large carnivores instigating trophic cascades is relatively weak (Allen et al. 2017; Blossey and Hare 2022), despite the widespread assumption that trophic cascades are commonplace in structuring food webs and ecological functioning (Pace et al. 1999; Schmitz et al. 2000; Halaj and Wise 2001). Demonstrating the structuring effects of large carnivores across free‐living systems is difficult, and the majority of studies published on trophic cascades have been critiqued for methodological flaws resulting in a lack of inferential power (Schmitz et al. 2000; Allen et al. 2013, 2017; Ford and Goheen 2015; Fleming 2019). These methodological challenges and lack of convincing evidence leave us with continued uncertainty regarding the ecological context that may enable large carnivores to cause a trophic cascade by exerting strong direct or indirect effects (Ford and Goheen 2015). Given the social and political controversy surrounding predator reintroductions and the growing interest in rewilding (Nogués‐Bravo et al. 2016; Blossey and Hare 2022), it is imperative to better understand if, how, and when large carnivore reintroductions can influence both forest regeneration and protection of other primary producers.

We investigated the existence of a potential trophic cascade triggered by a naturally expanding wolf population in northern Wisconsin, USA, and its ability to reverse negative deer impacts on woody and herbaceous plants. Wolves recolonized the state starting in the late 1970s and have expanded their range and population ever since (Wydeven et al. 2009; Fischer et al. 2022). This recolonization created a gradient of wolf density and residence time (i.e., years of wolf pack presence since recolonization) in a relatively sparsely human‐populated area of the state. We deployed a standardized sentinel protocol to assess deer browse severity (Blossey et al. 2019; Quirion and Blossey 2023) at 42 sites across this wolf gradient. By planting the same set of three species (white oak, *Quercus alba* ; red oak, *Quercus rubra* ; and zigzag goldenrod, *Solidago flexicaulis* ) at all sites, we were able to assess potential impacts of wolves across different forests. This approach enabled us to isolate potential wolf effects without interference by site‐specific differences in land‐use history and local plant community composition, which often make comparisons between sites difficult (Quirion and Blossey 2023). We hypothesized that deer browse on our sentinel plants would be reduced as a function of increased (1) wolf density and (2) wolf residence time. To further assess whether any potential wolf‐associated reduction in browse intensity would influence plant community composition over time, we recorded the presence of 11 common understory herbaceous plant species at each site. We expected that increased wolf residence time would create an extended legacy of protection from deer browse that would result in the recovery of herbaceous plants. We therefore hypothesized that (3) the probability of encountering individuals of 11 common understory plants at a site would increase as a function of increasing wolf residence time.

### 1.1 Ecological Setting

Today, mixed forests cover over 70% of northern Wisconsin (WDNR 2001). Our study focuses on hardwood forests, defined as any combination of sugar maple ( *Acer saccharum* ), beech ( *Fagus grandifolia* ), basswood (*Tilia americana*), white ash ( *Fraxinus americana* ), and yellow birch ( *Betula alleghaniensis* ) comprising more than 50% of the basal area (WDNR 2013). The most common associates are red maple ( *Acer rubrum* ), red oak ( *Quercus rubra* ), hemlock ( *Tsuga canadensis* ), white pine (*Pinus strobus*), and balsam fir ( *Abies balsamea* ). Northern hardwoods grow best on mesic sites (Table A1) with well‐drained to moderately well‐drained loamy soils and with soil pH ranging from 3.7–7.3, but a pH between 5.5 and 7.3 is most common.

**FIGURE 1:**
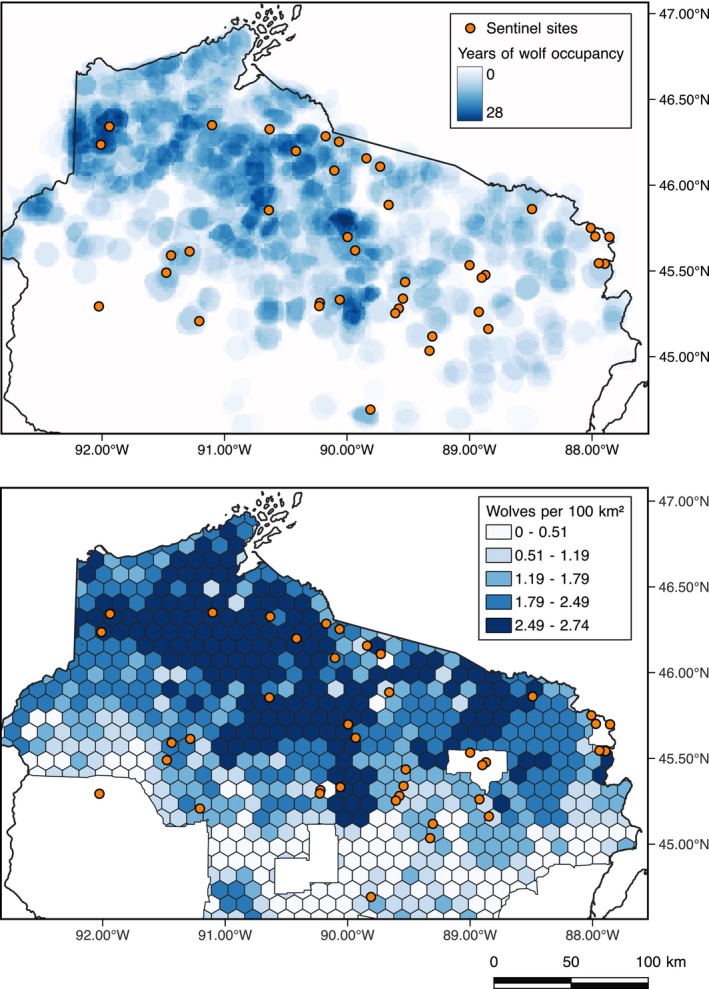
Wolf residence time (top, 1980–2019) and wolf density (bottom, winter 2020–2021) in relation to our study sites (orange points) in northern Wisconsin. Areas in the wolf density map that do not have hexagonal cells had no evidence of wolf activity for at least 4 years.

Following European settlement (~1830s), unregulated hunting, trapping, and bounties resulted in a collapse of the Wisconsin wolf population from an estimated 3000–5000 individuals to their extirpation from the state by 1960 (Wydeven et al. 2009). Protected by the Endangered Species Act (ESA), wolves recolonized northern Wisconsin from Minnesota in 1975, and the population steadily expanded from a few individuals to 1126 wolves in 292 packs by 2021 (Wydeven et al. 2009; McNamara et al. 2021). Wolves were delisted in January 2012 and, despite various court battles, several hundred wolves were legally killed in Wisconsin during the 2012–2014 hunting seasons until wolves once again came under ESA protection. Wolves were again briefly delisted from the ESA in 2020, resulting in a wolf hunt in 2021 that killed 218 individuals in just a few days (McNamara et al. 2021) before they were relisted in 2022. The majority of wolves reside in the northern third of the state (Figure 1), favoring habitats with more deciduous forest, less agricultural land, and low road density (Mladenoff et al. 2009). Pack sizes average 3.6 wolves with typical home ranges of 164.3 km^2^ (McNamara et al. 2021).

Deer (mostly fawns and older adults) account for the majority of wolf prey (DelGiudice et al. 2009), but berries and beaver ( *Castor canadensis* ) can be seasonally important (Fuller 1989), as can bait and gut piles left by bear hunters (Gable et al. 2018). Wisconsin's deer populations have steadily increased over the past decades and are at historic highs, with an estimated 1.4–1.7 million individuals statewide in 2021, and 376,000 in the northern portion of the state (Wojcik et al. 2021). Although estimates of deer abundance are not available at a local scale, estimated county‐level deer densities range from 2.7 to 17.8 deer/km^2^ (Wojcik et al. 2021), with densities as low as 4 deer/km^2^ able to prevent regeneration of many woody and herbaceous species in northern Wisconsin forests (Alverson et al. 1988).

## 2 Methods

We selected sites in northern Wisconsin (Figure 1) classified as “northern hardwood forests” from the Wisconsin Forest Inventory and Reporting System (WisFIRS). WisFIRS stores data collected by foresters and habitat managers on public lands (state or county forests) and tracks planned and completed treatments (e.g., timber sales, thinning, and prescribed burns). We selected sites classified as mesic with medium to rich soils (Table A1), > 12 ha in size, that had no management actions for at least 10 years and no planned treatments for the duration of our study. We then divided the known range of wolf density in this region (Figure 1) into six equal intervals, with the addition of a “no wolf” category (i.e., no wolves, 0.29–0.70 wolves/100 km^2^, 0.71–1.11 wolves/100 km^2^, 1.12–1.52 wolves/100 km^2^, 1.53–1.93 wolves/100 km^2^, 1.94–2.33 wolves/100 km^2^, and 2.34–2.75 wolves/100 km^2^), and randomly selected between 3 and 8 sites within each interval (median = 5 sites, dependent on the availability of suitable sites). Within our site selection, we were also able to create a gradient of wolf residence time (0–26 years, Figure 1). To increase the chance of independence among sites, we ensured that all sites were at least 2 km apart (approximate deer home range size in neighboring northern Michigan; Nielsen and Stroud‐Settles 2018) and that no sites were within the same 100‐km^2^ wolf occupancy hexagon published in the 2021 Wolf Monitoring Report (McNamara et al. 2021). Though we lack data on previous land‐use at our sites, including time of abandonment and successional dynamics, northern Wisconsin continues to be a working landscape with a history of logging and agriculture, and we expect that these activities previously occurred at our sites to varying degrees. Land abandonment and return to a forested landscape followed a typical pattern in that marginal lands with the poorest soil fertility could not compete in the agricultural economy and were abandoned first, but ultimately, agriculture was mostly abandoned throughout the region (Larson and Larson 2016; Yu and Lu 2018).

We followed the sentinel protocol (Blossey et al. 2017, 2019; Quirion and Blossey 2023) to assess browse of indicator species over time. We selected red oak, white oak, and zigzag goldenrod (hereafter “goldenrod”), which had been validated as reliable indicators of deer browse in previous studies in New York State (Blossey et al. 2017, 2019, Quirion and Blossey 2023). Red oak and goldenrod are widespread across Wisconsin, whereas white oak is uncommon in northern Wisconsin but part of Wisconsin Department of Natural Resources (WDNR) and US Forest Service assisted migration studies (Guries et al. 2019; Royo et al. 2023). We propagated seedlings in cone‐tainers (3.8 cm diameter × 14 cm deep; Stuewe and Sons, Tangent, OR, USA) using acorns collected in Wisconsin and goldenrod seeds purchased from Prairie Moon Nursery in Minnesota, following previously established methods (Blossey et al. 2017, 2019, Quirion and Blossey 2023).

From 3–15 June 2022, we established transects (50–100 m) at each of 40 sites. We planted 20 red oak seedlings (*N* = 800) at each site. Because of a shortage of sufficiently tall white oak seedlings, we planted at least 16 white oak seedlings at each of 23 sites (stratifying across wolf density, *N* = 368 seedlings). The distance between planting locations was 1 m, and we alternated red and white oaks at sites where both species were planted, skipping unsuitable planting locations (e.g., next to large trees or in windfalls). Using a handheld drill with a 2.5 cm diameter and 30 cm long masonry drill bit, we created tapered planting holes (10–15 cm deep × 5 cm wide). We removed seedlings as plugs from their cone‐tainers, removed the acorn to reduce rodent attraction, placed seedlings in the planting holes, and covered the potting soil with local soil. We staked a numbered metal tag (National Band and Tag Company, Newport, KY, USA) next to each seedling and recorded seedling height and number of leaves.

We revisited all sites once from 27 July to 5 August 2022, and recorded deer browse, rodent or insect attack, and seedling height. During this visit, we also measured the heights of the surrounding vegetation and litter depth (average of four points) within a 1 m^2^ quadrat centered on each seedling, visually estimated vegetation cover (%), and noted the presence/absence of non‐native earthworms at each site using the presence of castings and observation of individuals (sensu Dávalos, Simpson, et al. 2015). We collected this additional information as previous work demonstrated that these variables may affect seedling survival and growth, as well as deer browse preferences and severity (Blossey et al. 2019; Dobson et al. 2024). To explore potential cascading wolf effects on plant community composition, we recorded the presence of individuals of 11 common and widespread species every 10 m along each transect within a 5 m radius of the sampling point: *Aralia nudicaulis, Carex pensylvanica, Clintonia borealis, Eurybia macrophylla, Geranium maculatum, Hydrophyllum virginiana, Maianthemum canadense, Maianthemum racemosum, Pteridium aquilinum, Solidago flexicaulis*, and *Trillium grandiflorum*. These species represent a range of deer feeding preferences and browse sensitivities (Rogers et al. 1981) and, when taken as a portfolio, are a good indicator of the long‐term effects of deer and, consequently, wolves.

From 16–26 May 2023, we established new transects at 37 of our original sites following the same procedures as in 2022, with the addition of goldenrod as a sentinel. We replaced one site to more accurately match overstory composition at our other sites and replaced another because of timber harvest. Local foresters did not allow planting goldenrod at three sites. Our 2023 plantings included 20 goldenrods at each of 36 sites (*N* = 720) and 17–23 red oaks (*N* = 755) and white oaks (*N* = 788) at each of 39 sites. We revisited both transects at all sites from 22–31 August 2023 and 5–14 August 2024, recording the same data as in August 2022, except for plant community composition.

We used both wolf density and wolf residence time (Figure 1) to test for a potential top‐down trophic cascade. Wolf density was estimated for the winter of 2020–2021 across 100‐km^2^ hexagonal cells by WDNR staff using a scaled occupancy model built using data derived from winter track surveys and GPS‐collared wolves (McNamara et al. 2021; Stauffer et al. 2021). We derived the number of years a site was occupied by wolves since recolonization (i.e., wolf residence time) from overlapping annual maps of wolf pack locations from 1980, 1982, 1983, 1986, 1987, 1991–2010, and 2015–2019. These maps were created by WDNR by incorporating ground‐based snow tracking surveys, aerial observations, and location data from radio‐collared wolves to delineate pack areas and estimate pack size (McNamara et al. 2021). We obtained the maps from historic reports, georeferenced them within QGIS (QGIS Development Team 2023), and hand‐delineated territories. We buffered each territory by 1‐km to account for error in the georeferencing process. We estimated residence time as the number of years a site falls within a pack territory (0–26 years). Because of missing years of wolf pack maps and spatial shifts in pack territories, our definition of wolf residence time does not necessarily imply continuous presence.

### 2.1 Analyses

Our primary goal was to test for an association of deer browse rates and wolf density and wolf residence time, while accounting for other drivers known to affect plant survival, growth, and deer browse probabilities (Table 1). To test for this association, we selected a suite of candidate models on the basis of our original hypothesis that deer browse of our sentinel plants would decrease as a function of increased wolf density and wolf residence time. Alternatively, the null hypothesis is that neither wolf density nor wolf residence time is associated with deer browse rates in northern Wisconsin. The model set included models with and without the wolf predictors, as well as models with an interaction between wolves and covariates that may represent forage availability (e.g., precipitation, vegetation cover, and quadratic mean diameter; see below). We included these interactions to account for the vast complexity inherent in food web dynamics. We selected the best model from the suite using AICc and considered models within 2 AICc points of the best model to perform nearly as well.

**TABLE 1:** Environmental predictor variables used in models of deer browse, sentinel growth, and understory plant community composition, and supporting references.

Predictor	Predicted effect	References
Planting height	Browse: Taller plants have a higher probability of being detected and browsed by deer	Brice et al. (2022, 2024)
	Growth: Taller plants could grow faster because of their genotype, increased resource allocation to above‐ground growth, improved resource capture, or competitive ability for light	Rounds (1979), Motta (2003), Renaud et al. (2003), Konôpka et al. (2018), Maxwell et al. (2019)
Leaf litter	Growth: Reduced litter depth may indicate earthworm presence and faster nutrient cycling, leading to increased plant growth (a fertilization effect). In contrast, increased leaf litter may allow improved mycorrhizal colonization, reduced evaporation and erosion, and better buffering of soil temperature, which can all improve plant growth	Dobson et al. (2017, 2020, 2024), Frelich et al. (2019)
	Plant community: The effects at the individual plant level, as listed for growth, may allow certain species to respond differently, and thus, litter depth may affect the frequency of occurrence of the 11 common species. The direction of this response could be an increase or a decrease, depending on the specific plant species	Craven et al. (2017), Nuzzo et al. (2017), Dobson et al. (2024)
Earthworm presence	Browse: Because of the positive association between non‐native earthworm presence and deer abundance, plus the fertilization effect of earthworms, we expect increased browsing at sites with non‐native earthworms	Dobson and Blossey (2015), Blossey et al. (2017), Dobson et al. (2020, 2024)
	Plant community: Non‐native worms may impact the plant community through their effects on soil microbes, mycorrhizal associations, nutrient cycling, leaf litter depth, and so forth. The direction of this response could be an increase or a decrease depending on the specific plant species	Dávalos, Nuzzo, and Blossey (2015), Craven et al. (2017), Frelich et al. (2019), Reed et al. (2023)
Vegetation cover	Browse: Increased plant cover around a planted seedling will reduce detection by deer, or provide alternative forage, suggesting a decrease in browse probability as cover increases	Bee et al. (2009), Champagne et al. (2016), Blossey et al. (2017), Royo et al. (2017)
	Growth: Growth of planted individuals is expected to decrease as surrounding vegetation cover increases because of higher resource competition	Craine and Dybzinski (2013)
	Plant community: At sites with more vegetative cover, the frequency of common understory plants is expected to increase because of generally higher plant abundance	Rogers et al. (2009), Frerker et al. (2014), Karbstein et al. (2023)
Summer precipitation	Browse: Sites with greater average summer precipitation avoid water limitation and produce more forage available to deer, which is expected to reduce browse on planted seedlings	McEwan et al. (2011), Craine and Dybzinski (2013)
	Growth: Seedling growth increases with increasing summer precipitation because of an increase in water availability	Craine and Dybzinski (2013)
	Plant community: Common plants are more likely to be found at sites with more summer precipitation because of an increase in water availability	Craine and Dybzinski (2013)
Quadratic mean diameter (QMD)	Browse: A higher QMD indicates less light available in the understory, which reduces forage availability for deer and increases browsing of planted seedlings Growth: Sites with increased QMD will have less abundant growth of understory species because of light limitation Plant community: At high QMD, light limitation will reduce the frequency of the 11 common understory plant species	Comeau and Heineman (2003)

Though we are primarily interested in testing for a wolf‐driven trophic cascade, we also included several bottom‐up variables that are known to influence deer browsing rates (Table 1). Including these additional variables was necessary to avoid confounding a wolf effect on vegetation that instead could be driven by abiotic or biotic site characteristics (e.g., Romme et al. 2005). As such, all models included planting height (cm), vegetation cover (%) in 1‐m^2^ around each seedling, non‐native earthworm presence/absence, quadratic mean diameter of trees at each site (QMD, cm), and summer precipitation (mm) from the previous year. We also included a fixed effect for species in oak models. We obtained QMD for each site from WisFIRS and estimated previous summer precipitation as average monthly precipitation from May–September at each site using daily climate data from Daymet (Thornton et al. 2020). We collected all other variables in situ.

We built generalized linear mixed models (GLMM) fit with a binomial error distribution using the lme4 package in R (Bates et al. 2015). We analyzed browse on oaks (*N* = 2731) separately from browse on goldenrod (*N* = 695) because of different monitoring time frames: oaks included data from two summers (June 2022–Aug 2023 for transect 1, May 2023–Aug 2024 for transect 2) because of insufficient browse events in the first year, and goldenrod included data from a single season (May 2023–Aug 2023). We scaled all continuous predictors to have a mean of zero and standard deviation of one so that the strength of predictors could be compared and included a random intercept for site. The oak models also included a random intercept for transect (i.e., planted in 2022 vs. 2023) nested within site. We tested for multicollinearity using the *vif* function in the car package (Fox and Weisberg 2019), assessed model fit using the DHARMa package (Hartig 2021), and plotted model predictions with the emmeans package (Lenth 2023).

To further investigate the potential effects of wolves on forest regeneration and understory plant performance, we compared a suite of plant growth models to test whether wolf density and wolf residence time are positively associated with plant growth. We again included environmental variables that could affect plant growth and survival (Table 1) and tested for interactions between these variables and wolves and browsing. For oaks, we modeled changes in the height of seedlings planted in June 2022 that were still present in August 2024 (*n* = 890; exposed to deer for three summers). As goldenrods are a perennial that regrow new above‐ground tissues each year, we modeled change in height for individuals planted in May 2023 that were still present in August 2023 (*n* = 686; exposed to deer for one summer). As with browsing, we modeled oaks and goldenrod separately.

Despite a slight negative correlation between wolves and deer browse (−0.15 to −0.19), we felt it prudent to include both covariates in the growth models. Wolf density and residence time represent data collected at a fairly coarse spatial scale (e.g., > 100 km^2^), whereas deer browse may capture nuances of the impact of wolves at a much finer scale (e.g., wolf space use within a territory that is impacting browse). All models included the following variables expected to influence plant growth: planting height (cm), leaf litter depth (cm), vegetation cover (%), summer precipitation (mm), and QMD (cm). We included a fixed effect for species in the oak models and a binary predictor for other plant damage (e.g., rodent cuts, tip death, and unknown damage). There were too few cases of “other damage” (*n* = 20) to include this predictor in the goldenrod models. We modeled growth with a Normal distribution and assessed model fit as previously described.

Finally, we explored the relationship between wolves and the understory plant community by comparing a suite of models for the presence of 11 common understory plants. The model suite included models with and without deer browse, wolf density, and wolf residence time. We again tested for interactions that may capture the complexity of food web dynamics, namely interactions between plant species and deer browse, wolves, and QMD. All models included plant species and several other site characteristics (Table 1). We modeled the probability of plant species presence at each site using a GLMM fit with a binomial error distribution (successes = number of sampling points at which a species was observed along the transect, failures = number of sampling points at which the species was not observed along the transect). We included a random intercept for site and scaled all continuous predictors to have a mean of zero and a standard deviation of one.

## 3 Results

For deer browse probability, the best‐approximating model included a main effect of wolf residence time for oaks and an interaction between wolf density and vegetation cover for goldenrod (Tables 2, 3, A2). Both models were a better fit for the data than the null models by more than 2 AICc points, suggesting we reject the null hypothesis that wolves do not affect deer browse. A model with wolf density rather than wolf residence time was within 2 AICc of the best oak model, and the model with an interaction between wolf residence time and vegetation cover was within 2 AICc of the best goldenrod model (Tables 2 and 3). Oak models with an interaction between wolf residence time and precipitation, QMD, and vegetation cover and wolf density and precipitation were also within 2 AICc of the best model (Table 2). These results suggest the possibility of complex trophic interactions that include other abiotic and biotic drivers, but in the following, we will discuss only the best‐approximating model, which is also the most parsimonious.

**TABLE 2:** Candidate model set results used to determine the best‐approximating model describing the probability of deer browse of red and white oak across a gradient of wolf density and residence time in northern Wisconsin.

Model structure	K	AIC_C_	Δ AIC_C_	W_i_
Oak species + planting height + vegetation cover + earthworms + precipitation + QMD + wolf time	10	1971.21	0.00	0.24
Oak species + planting height + vegetation cover + earthworms + precipitation + QMD + wolf density + wolf density*precipitation	11	1971.97	0.76	0.17
Oak species + planting height + vegetation cover + earthworms + precipitation + QMD + wolf time + wolf time*vegetation cover	11	1972.31	1.11	0.14
Oak species + planting height + vegetation cover + earthworms + precipitation + QMD + wolf time + wolf time*precipitation	11	1972.52	1.31	0.13
Oak species + planting height + vegetation cover + earthworms + precipitation + QMD + wolf time + wolf time*QMD	11	1972.93	1.72	0.1
Oak species + planting height + vegetation cover + earthworms + precipitation + QMD + wolf density	10	1973.03	1.82	0.1
Oak species + planting height + vegetation cover + earthworms + precipitation + QMD	9	1974.29	3.09	0.05
Oak species + planting height + vegetation cover + earthworms + precipitation + QMD + wolf density + wolf density*vegetation cover	11	1974.87	3.67	0.04
Oak species + planting height + vegetation cover + earthworms + precipitation + QMD + wolf density + wolf density*QMD	11	1974.97	3.76	0.04

*Note:* AICc = Corrected Akaike's Information Criterion; ∆AICc = relative difference from the best model; K = Number of parameters; Wi = Akaike weight.

**TABLE 3:** Candidate model set results used to determine the best‐approximating model describing the probability of deer browse of goldenrod across a gradient of wolf density and residence time in northern Wisconsin.

Model structure	K	AIC_C_	Δ AIC_C_	W_i_
Planting height + vegetation cover + earthworms + precipitation + QMD + wolf density + wolf density*vegetation cover	9	749.51	0.00	0.71
Planting height + vegetation cover + earthworms + precipitation + QMD + wolf time + wolf time*vegetation cover	9	751.33	1.82	0.28
Planting height + vegetation cover + earthworms + precipitation + QMD + wolf density	8	760.49	10.98	0
Planting height + vegetation cover + earthworms + precipitation + QMD	7	761.71	12.20	0
Planting height + vegetation cover + earthworms + precipitation + QMD + wolf time	8	762.07	12.56	0
Planting height + vegetation cover + earthworms + precipitation + QMD + wolf density + wolf density*precipitation	9	762.47	12.96	0
Planting height + vegetation cover + earthworms + precipitation + QMD + wolf density + wolf density*QMD	9	762.54	13.03	0
Planting height + vegetation cover + earthworms + precipitation + QMD + wolf time + wolf time*precipitation	9	764.05	14.54	0
Planting height + vegetation cover + earthworms + precipitation + QMD + wolf time + wolf time*QMD	9	764.12	14.61	0

*Note:* AICc = Corrected Akaike's Information Criterion; ∆AICc = relative difference from the best model; K = number of parameters; Wi = Akaike weight.

**FIGURE 2:**
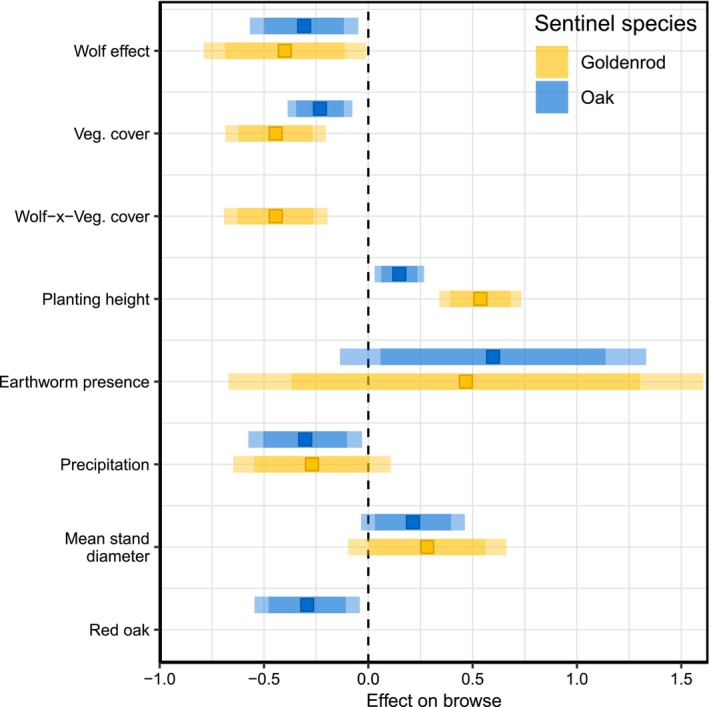
Coefficients from logistic regression of browse probability for white and red oak (blue) and goldenrod (yellow). Estimates are shown for the best model of each dataset. Squares show parameter estimates (log‐odds), darker error bars show 85% confidence intervals and lighter error bars show 95% confidence intervals. The dashed vertical line indicates zero effect. “Wolf effect” represents wolf residence time in the oak model and wolf density in the goldenrod model. In the goldenrod model, wolf density interacts with vegetation cover (denoted by the “Wolf‐x‐Veg. cover” parameter). The parameter “red oak” represents the difference in the log‐odds of browsing between red oak and white oak (the reference group). All continuous predictors were scaled to have a mean of 0 and standard deviation of 1.

**FIGURE 3:**
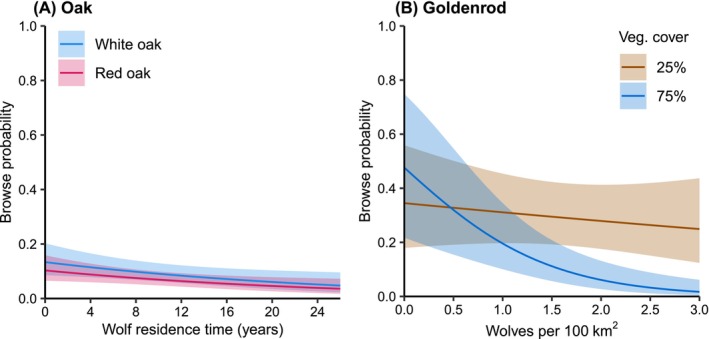
Predicted effects of (A) wolf residence time on oak browse probability and (B) wolf density on goldenrod browse probability at low (25%) and high (75%) vegetative cover. Shaded areas are 95% confidence intervals. Predictions were made with all continuous predictors set to their mean values, and averaged across levels of categorical predictors. Mean values in oak model: Planting height = 12.8 cm, vegetation cover = 39.2%, summer precipitation = 83.8 mm, quadratic mean diameter = 29.5 cm. Goldenrod model: Planting height = 19.9 cm, summer precipitation = 86.8 mm, quadratic mean diameter = 29.3 cm.

For red and white oak, deer browse probabilities (β = −0.3, 95% Confidence Interval = −0.6, −0.05, Table 4, Figure 2) decreased 4.1% (95% CI = 7.6% to 0.7%) for every 1‐year increase in wolf residence time (Figure 3a). For example, browse probability for a white oak seedling in the absence of wolves is 0.134 and decreases to 0.108 at sites with 10 years of wolf residence time. For goldenrod, vegetative cover affected the strength of the wolf effect on deer browse probability: as vegetation cover increased, the protective effect of wolves on seedlings became stronger (Main effect β = −0.4, 95% CI = −0.8, −0.02; Interaction β = −0.4, 95% CI = −0.7, −0.2, Table 5). For example, with an increase from 0 to 1 wolf/100 km^2^, average predicted browse probability on goldenrods decreases from 0.34 to 0.31 with 25% vegetative cover, but from 0.48 to 0.20 with 75% vegetative cover (Figure 3b).

**TABLE 4:** Parameter estimates, standard errors (SE), *z*‐values, and *p*‐values for the top supported models (as determined by AICc scores; see Tables 2 and 3) describing the probability of oak and goldenrod being browsed by deer in northern Wisconsin.

Parameter	Estimate	SE	z‐value	Pr(>|z|)
Red and white oak				
Intercept	−2.52	0.36	−7.07	< 0.001
Species: *Q. rubra*	−0.29	0.13	−2.28	0.02
Planting height	0.15	0.06	2.46	0.01
% vegetation cover	−0.23	0.08	−2.93	< 0.001
Earthworm presence	0.60	0.37	1.60	0.11
Summer precipitation	−0.30	0.14	−2.17	0.03
Quadratic mean diameter	0.21	0.13	1.69	0.09
Wolf residence time	−0.31	0.13	−2.32	0.02
Goldenrod				
Intercept	−1.35	0.54	−2.52	0.01
Planting height	0.54	0.10	5.34	< 0.001
% vegetation cover	−0.44	0.12	−3.61	< 0.001
Earthworm presence	0.47	0.58	0.81	0.42
Summer precipitation	−0.27	0.19	−1.40	0.16
Quadratic mean diameter	0.28	0.19	1.46	0.14
Wolf density	−0.40	0.20	−2.01	0.04
Wolf density × Veg. cover	−0.44	0.13	−3.50	< 0.001

*Note:* For species, the reference group is *Q. alba*; the parameter “Species: *Q. rubra* ” represents the difference in the main effect of *Q. alba* and *Q. rubra* . All predictors were scaled to have a mean of zero and a standard deviation of 1, meaning that coefficients represent the change in the log‐odds of the response for a 1 standard deviation change in the predictor.

**TABLE 5:** Candidate model set results used to determine the best‐approximating model describing the height increase of red and white oak across a gradient of wolf density and residence time in northern Wisconsin.

Model structure	K	AIC_C_	Δ AIC_C_	W_i_
Oak species + deer browse + other damage + planting height + precipitation + litter depth + veg cover + QMD + wolf density + deer browse*wolf density	13	5154.98	0.00	0.58
Oak species + deer browse + other damage + planting height + precipitation + litter depth + veg cover + QMD + wolf time + deer browse*wolf time	13	5157.51	2.54	0.16
Oak species + deer browse + other damage + planting height + precipitation + litter depth + veg cover + QMD	11	5158.8	3.83	0.09
Oak species + deer browse + other damage + planting height + precipitation + litter depth + veg cover + QMD + wolf density	12	5160.13	5.15	0.04
Oak species + deer browse + other damage + planting height + precipitation + litter depth + veg cover + QMD + wolf time	12	5160.46	5.48	0.04
Oak species + deer browse + other damage + planting height + precipitation + litter depth + veg cover + QMD + wolf density + deer browse*litter depth	13	5161.78	6.81	0.02
Oak species + deer browse + other damage + planting height + precipitation + litter depth + veg cover + QMD + wolf density + deer browse*precipitation	13	5162.12	7.14	0.02
Oak species + deer browse + other damage + planting height + precipitation + litter depth + veg cover + QMD + wolf time + deer browse*litter depth	13	5162.13	7.15	0.02
Oak species + deer browse + other damage + planting height + precipitation + litter depth + veg cover + QMD + wolf density + deer browse*QMD	13	5162.19	7.21	0.02
Oak species + deer browse + other damage + planting height + precipitation + litter depth + veg cover + QMD + wolf time + deer browse*precipitation	13	5162.46	7.49	0.01
Oak species + deer browse + other damage + planting height + precipitation + litter depth + veg cover + QMD + wolf time + deer browse*QMD	13	5162.52	7.54	0.01

*Note:* AICc = Corrected Akaike's Information Criterion; ∆AICc = relative difference from the best model; K = number of parameters; Wi = Akaike weight.

**FIGURE 4:**
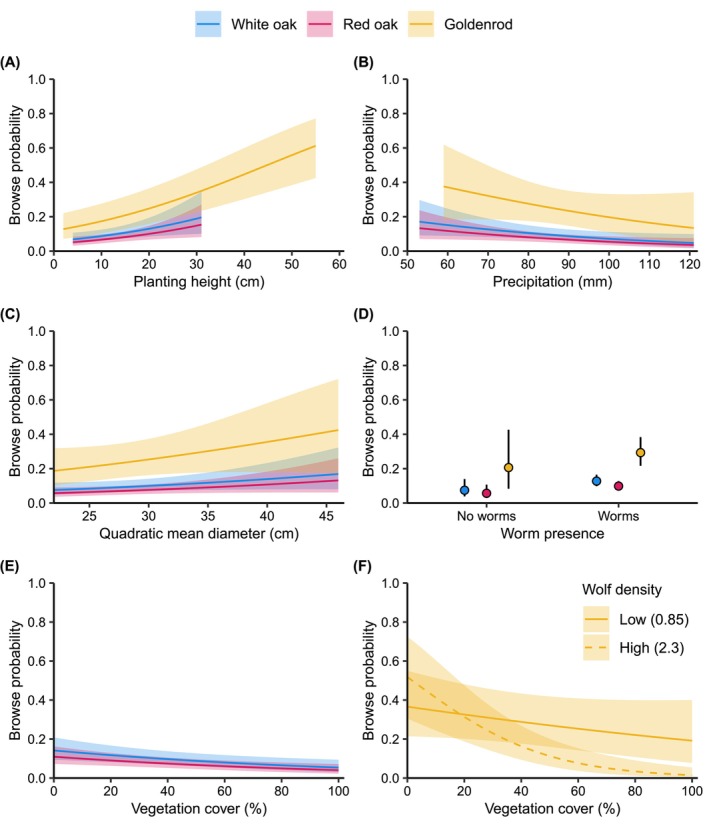
Predicted effects and 95% confidence intervals of (A) planting height, (B) summer precipitation, (C) quadratic mean tree diameter, (D) worm presence, and (E, F) vegetation cover on browse probability of sentinel species. (E) shows vegetation cover for white and red oak, and (F) shows vegetation cover interacting with wolf density for goldenrod. All predictions are from the best‐approximating models of oak and goldenrod browsing. Predictions are limited to the observed values for each species, and were made with all continuous covariates, excluding the focal covariate, set to their mean values, and averaged across categorical variables. Mean values in oak model: Planting height = 12.8 cm, vegetation cover = 39.2%, summer precipitation = 83.8 mm, QMD = 29.5 cm, wolf residence time = 8.1 years. Goldenrod model: Planting height = 19.9 cm, vegetation cover = 37.1%, summer precipitation = 86.8 mm, QMD = 29.3 cm.

Browse probability increased as planting height and QMD increased or in the presence of earthworms, whereas precipitation and increased vegetative cover had the opposite effect, with varying degrees of uncertainty for oak and goldenrod (Table 4, Figures 2 and 4). White oak was 1.3 times more likely to be browsed than red oak (Table 4, Figure 2). Non‐native earthworm presence was one of the strongest drivers of deer browse for all sentinel species, but also had the most uncertainty in parameter estimates (Figures 2 and 4).

For both oak and goldenrod growth, the best‐approximating models included an interaction between wolf density and deer browse (Tables 5, 6, A2). The null model in both cases was more than 2 AICc points greater than the best model, suggesting we reject the null hypothesis that wolves do not affect plant growth. No other model was within 2 points of the best oak model, but the model including wolf density and an interaction between deer browse and QMD was within 2 points of the best goldenrod model (Tables 5 and 6).

**TABLE 6:** Candidate model set results used to determine the best‐approximating model describing the height increase of goldenrod across a gradient of wolf density and residence time in northern Wisconsin.

Model structure	K	AIC_C_	Δ AIC_C_	W_i_
Deer browse + other damage + planting height + precipitation + litter depth + veg cover + QMD + wolf density + deer browse*wolf density	11	4825.38	0	0.47
Deer browse + other damage + planting height + precipitation + litter depth + veg cover + QMD + wolf density + deer browse*QMD	11	4827.31	1.92	0.18
Deer browse + other damage + planting height + precipitation + litter depth + veg cover + QMD + wolf density + deer browse*precipitation	11	4827.84	2.46	0.14
Deer browse + other damage + planting height + precipitation + litter depth + veg cover + QMD + wolf time + deer browse*QMD	11	4829.39	4.01	0.06
Deer browse + other damage + planting height + precipitation + litter depth + veg cover + QMD + wolf time + deer browse*precipitation	11	4829.61	4.23	0.06
Deer browse + other damage + planting height + precipitation + litter depth + veg cover + QMD + wolf density	10	4831.07	5.68	0.03
Deer browse + other damage + planting height + precipitation + litter depth + veg cover + QMD + wolf time + deer browse*wolf time	11	4831.56	6.18	0.02
Deer browse + other damage + planting height + precipitation + litter depth + veg cover + QMD	9	4831.65	6.26	0.02
Deer browse + other damage + planting height + precipitation + litter depth + veg cover + QMD + wolf density + deer browse*litter depth	11	4832.85	7.46	0.01
Deer browse + other damage + planting height + precipitation + litter depth + veg cover + QMD + wolf time	10	4833.02	7.64	0.01
Deer browse + other damage + planting height + precipitation + litter depth + veg cover + QMD + wolf time + deer browse*litter depth	11	4834.76	9.37	0

*Note:* AICc = Corrected Akaike's Information Criterion; ∆AICc = relative difference from the best model; K = number of parameters Wi = Akaike weight.

**FIGURE 5:**
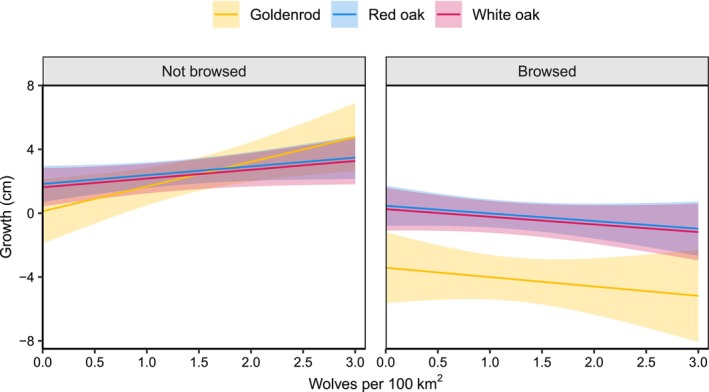
Predicted effect of wolf density on seedling growth for unbrowsed individuals (left panel) versus those browsed by deer (right panel). Oak growth was measured over 3 summers, and goldenrod growth was measured after 1 summer. Shaded ribbons represent 95% confidence intervals. Predictions were made with all continuous covariates set to their mean values and averaged across categorical variables. Mean values in oak model: Planting height = 13.0 cm, leaf litter = 1.3 cm, vegetation cover = 39.7%, summer precipitation = 84.4 mm, quadratic mean diameter = 29.4 cm. Goldenrod model: Planting height = 19.7 cm, leaf litter = 1.1 cm, vegetation cover = 37.1%, summer precipitation = 86.8 mm, quadratic mean diameter = 29.3 cm.

The effect of wolves on seedling growth was affected by whether sentinel plants were browsed (Table 7, Figure 5). As wolf density increased, the growth of unbrowsed seedlings increased, but declined for browsed seedlings. This interaction was stronger for goldenrod (β = −2.0, 95% CI = −3.3, −5.8) than for oak (β = −0.9, 95% CI = −1.6, −2.8). For example, the height of unbrowsed oaks increased 0.6 cm with an increase of 1 wolf/100 km^2^, but browsed oaks decreased by 0.5 cm (Figure 5). Unbrowsed goldenrod individuals grew 1.6 cm taller, but browsed individuals lost 0.6 cm, with an increase of 1 wolf/100 km^2^ (Figure 5). Oak growth also decreased in the presence of other damage and with increased planting height, but oak growth increased as vegetation cover increased (Table 7). In contrast, goldenrods that were taller at planting grew more, but vegetative cover had no effect on their growth (Table 7).

**TABLE 7:** Parameter estimates, standard errors (SE), degrees of freedom (DF), *t*‐values, and *p*‐values for the top supported models (as determined by AICc scores; see Tables 5 and 6) describing the height growth of oak and goldenrod in northern Wisconsin.

Parameter	Estimate	SE	DF	*t*‐value	Pr(>|*t*|)
Red and white oak					
Intercept	3.47	0.35	121.0	9.95	< 0.001
Species: *Q. rubra*	0.21	0.34	878.0	0.62	0.54
Deer browse	−2.61	0.35	887.4	−7.43	< 0.001
Other damage	−2.38	0.61	885.8	−3.92	< 0.001
Planting height	−2.24	0.16	889.7	−13.86	< 0.001
% vegetation cover	1.08	0.19	363.7	5.82	< 0.001
Leaf litter depth	0.07	0.17	616.2	0.40	0.69
Precipitation	0.13	0.26	37.8	0.51	0.61
QMD	0.31	0.25	39.0	1.25	0.22
Wolf density	0.49	0.27	50.3	1.83	0.07
Deer browse × Wolf density	−0.92	0.34	823.4	−2.69	0.01
Goldenrod					
Intercept	2.28	0.50	52.8	4.59	< 0.001
Deer browse	−6.51	0.73	673.3	−8.95	< 0.001
Planting height	0.90	0.32	676.6	2.80	0.01
% vegetation cover	−0.16	0.36	399.2	−0.46	0.65
Leaf litter depth	−0.22	0.34	566.1	−0.65	0.52
Precipitation	0.61	0.45	37.4	1.36	0.18
QMD	0.40	0.46	35.3	0.88	0.38
Wolf density	1.42	0.52	57.2	2.74	0.01
Deer browse × Wolf density	−1.95	0.69	649.5	−2.82	< 0.001

*Note:* For species, the reference group is *Q. alba*; the parameter “Species: *Q. rubra* ” represents the difference in the main effect of *Q. alba* and *Q. rubra* . The reference groups for deer browse and other damage were 0 (no browse/damage). All predictors were scaled to have a mean of zero and a standard deviation of 1, meaning that coefficients represent the change in height for a 1 standard deviation change in the predictor.

**FIGURE 6:**
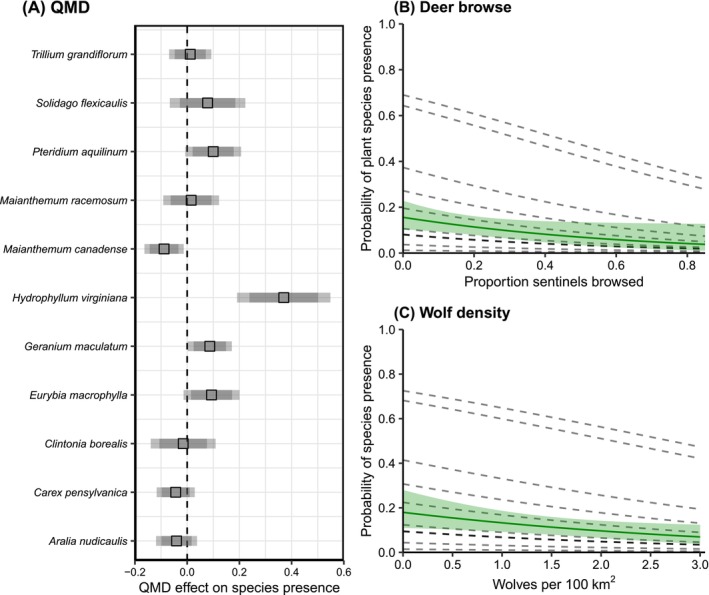
The effect of (A) forest structure (QMD), (B) deer browse, and (C) wolf density on the presence of 11 common understory plant species. (A) Adjusted coefficients for the effect of QMD on each of the 11 species: Squares show parameter estimates (log‐odds), darker error bars show 85% confidence intervals and lighter error bars show 95% confidence intervals. The dashed vertical line indicates zero effect. Green lines in (B) and (C) represent the average effect of deer and wolves on the presence of 11 common understory species, with green ribbons representing 95% confidence intervals. Dashed gray lines show predictions for individual species.

Deer browse, wolf density, and an interaction between plant species and QMD (Tables 8 and A3) were all included in the best model assessing the plant community effects. We found a negative effect of deer browse on the frequency of common understory plants (β = −0.3, 95% CI = −0.7, −0.01). For example, the probability of encountering a species at a site decreased by 83% as deer browse increased from 0 to 100% (Table 9, Figure 6). An increase in wolf density decreased the probability of encountering common plant species (β = −0.3, 95% CI = −0.6, −0.05). Every increase of 1 wolf/100 km^2^ (Table 9, Figure 6) decreased the probability of encountering one of our common plant species by 29.5%.

**TABLE 8:** Candidate model set results used to determine the best‐approximating model describing the presence of 11 common understory plants across a gradient of wolf density and residence time in northern Wisconsin.

Model structure	K	AIC_C_	Δ AIC_C_	W_i_
Plant species + veg. cover + earthworms + litter depth + precipitation + QMD + deer browse + wolf density + Species*QMD	29	964.24	0	0.83
Plant species + veg. cover + earthworms + litter depth + precipitation + QMD + deer browse + wolf time + Species*QMD	29	967.36	3.11	0.17
Plant species + veg. cover + earthworms + litter depth + precipitation + QMD + deer browse + wolf density + Species*wolf density	29	996.09	31.85	0
Plant species + veg. cover + earthworms + litter depth + precipitation + QMD + deer browse + wolf density	19	996.27	32.03	0
Plant species + veg. cover + earthworms + litter depth + precipitation + QMD + deer browse + wolf density + Species*deer browse	29	996.43	32.19	0
Plant species + veg. cover + earthworms + litter depth + precipitation + QMD	17	996.59	32.35	0
Plant species + veg. cover + earthworms + litter depth + precipitation + QMD + deer browse	18	997.78	33.54	0
Plant species + veg. cover + earthworms + litter depth + precipitation + QMD + deer browse + wolf time + Species*deer browse	29	998.82	34.57	0
Plant species + veg. cover + earthworms + litter depth + precipitation + QMD + deer browse + wolf time	19	998.83	34.59	0
Plant species + veg. cover + earthworms + litter depth + precipitation + QMD + deer browse + wolf time + Species*wolf time	29	1000.35	36.11	0

*Note:* AICc = Corrected Akaike's Information Criterion; ∆AICc = relative difference from the best model; K = number of parameters; Wi = Akaike weight.

**TABLE 9:** Parameter estimates, standard errors (SE), *z*‐values, and *p*‐values for the top supported model (as determined by AICc scores; see Table 8) describing the presence of 11 common understory plants in northern Wisconsin.

Parameter	Estimate	SE	*z*‐value	Pr(>|z|)
Intercept	−0.60	0.38	−1.57	0.12
Species: *Carex pensylvanica*	1.32	0.26	5.13	< 0.001
Species: *Clintonia borealis*	−1.90	0.38	−5.02	< 0.001
Species: *Eurybia macrophylla*	−1.91	0.39	−4.86	< 0.001
Species: *Geranium maculatum*	−0.89	0.30	−2.98	< 0.001
Species: *Hydrophyllum virginiana*	−3.87	0.90	−4.32	< 0.001
Species: *Maianthemum canadense*	1.11	0.26	4.31	< 0.001
Species: *Maianthemum racemosum*	−1.61	0.35	−4.63	< 0.001
Species: *Pteridium aquilinum*	−1.93	0.40	−4.85	< 0.001
Species: *Solidago flexicaulis*	−2.74	0.53	−5.19	< 0.001
Species: *Trillium grandiflorum*	−0.47	0.27	−1.70	0.09
Vegetation cover (%)	0.12	0.14	0.81	0.42
Earthworm presence	−0.45	0.38	−1.17	0.24
Leaf litter depth	0.10	0.16	0.62	0.54
Summer precipitation	−0.01	0.14	−0.08	0.93
Quadratic mean diameter (QMD)	−0.23	0.23	−1.02	0.31
Deer browse	−0.33	0.17	−1.99	0.05
Wolf density	−0.33	0.14	−2.28	0.02
*Carex pensylvanica* × QMD	−0.02	0.26	−0.07	0.94
*Clintonia borealis* × QMD	0.14	0.39	0.37	0.71
*Eurybia macrophylla* × QMD	0.76	0.34	2.21	0.03
*Geranium maculatum* × QMD	0.72	0.29	2.52	0.01
*Hydrophyllum virginiana* × QMD	2.33	0.54	4.32	< 0.001
*Maianthemum canadense* × QMD	−0.27	0.27	−1.02	0.31
*Maianthemum racemosum* × QMD	0.32	0.34	0.92	0.36
*Pteridium aquilinum* × QMD	0.79	0.34	2.31	0.02
*Solidago flexicaulis* × QMD	0.67	0.44	1.52	0.13
*Trillium grandiflorum* × QMD	0.30	0.28	1.07	0.29

*Note:* For species, the reference group is *Auralia nudicaulis*. All predictors were scaled to have a mean of zero and a standard deviation of 1, meaning that coefficients represent the change in the log odds of a species being present with a 1 standard deviation change in the predictor.

The effect of QMD on plant frequency varied by species, with the strongest positive association between QMD and *Hydrophyllum virginiana*, and the strongest negative association with *Maianthemum canadense* (Table 9, Figure 6). On average, a 1‐cm increase in QMD increased the probability of encountering *H. virginiana* by 43.7% (95% CI = 20.5, 71.4), but decreased the probability of encountering *M. canadense* by 8.2% (95% CI = 14.8, 1.1). The other variables included in the model were not associated with plant species presence (Table 9).

## 4 Discussion

Our predictions of an existing or developing trophic cascade associated with the return of wolves to northern Wisconsin were not unequivocally supported by the evidence we collected. We detected a pattern of decreased browse probability as wolf density and residence time increased, but wolf effects interacted with other biotic and abiotic factors that appeared equally strong in affecting deer browse probabilities on our sentinel plants. Furthermore, opposite to what would be expected if the return of wolves and their increase in density caused a trophic cascade, the frequency of 11 herbaceous plants declined as wolf density increased. We found the strongest effect of wolves in reducing deer browse at sites with existing high vegetative cover and tall understory plants (Figures 3 and 4). Our results suggest that wolf reintroduction alone would not be effective at restoring ecosystems that have a history of high deer browse pressure and low abundance of native understory species. Our findings also suggest the possibility that wolf effects may be powerful enough to help maintain ecosystems with abundant and thriving native plant species. However, we need additional experimental evidence to affirm these benefits to forest regeneration or herbaceous plant demography. Our findings echo recently published evidence that wolf effects on willow and aspen in Yellowstone National Park (Brice et al. 2024; Hobbs et al. 2024) were weaker than previously reported, and that bottom‐up factors, such as water availability, were important drivers of plant growth.

Our results are contradictory to some earlier work claiming the rapid development of a trophic cascade with the return of wolves to northern Wisconsin (Bouchard et al. 2013; Callan et al. 2013; Flagel et al. 2016). These previous studies compared understory plant growth, cover, species richness, and browse intensity in areas with high or low wolf activity or residence time. None of these studies, however, measured other factors that may explain species richness, plant size, or performance differences in herbaceous plants or shrubs at their research sites or used multiple regression to account for other potential drivers. The composition of plant communities is determined by a myriad of abiotic and biotic factors that include, but are not limited to: climate, precipitation, soils and soil nutrient availability, light availability, hydrology, previous land‐use, and presence of mutualists, competitors, and natural enemies. These factors then interact with species traits to act as filters on the regional species pool, thereby determining local plant community composition (Keddy 1992; Gerhardt and Foster 2002; Silvertown 2004; Flinn and Vellend 2005; Geho et al. 2007). Variation among sites in abiotic and biotic filters, dispersal limitations, and other forces creates heterogeneous landscapes—thus, we cannot assume that all hardwood forests in the region had similar plant species diversity, species composition, or size distribution before wolves returned. The differences in plant community richness or plant size between high and low wolf use areas can only be attributed to wolves if studies are able to control for other potential explanatory factors; otherwise, it can lead to a spurious wolf effect. Romme et al. (2005) found such an effect in Yellowstone: without considering soil pH, it appeared that aspen seedlings were taller near wolf dens, but this wolf effect was no longer significant when soil pH and available potassium were included in their models. Herein lies the challenge of documenting cascading effects of large carnivores in free‐living landscapes: correlation does not always imply causation.

Furthermore, wolves and their prey choose where to settle, give birth, and forage on the basis of food availability and quality and their own safety. For example, in northern Michigan (Potvin et al. 2005, O'Neil et al. 2019) and in Europe (Roder et al. 2020), dispersing wolves established territories primarily as a function of prey abundance (deer or other ungulates) and distance to or density of roads. Similarly, deer respond to variations in food availability and quality across landscapes and adjust their home ranges and forage patterns accordingly (Massé and Côté 2013; Muhly et al. 2013). Thus, it is possible that the patterns of increased plant species richness and growth in high wolf use areas reported by others (Bouchard et al. 2013, Callan et al. 2013, Flagel et al. 2016) were pre‐existing conditions favored by deer because of the high‐quality forage and subsequently favored by wolves because of higher deer abundance. This “chicken and egg” problem can only be resolved if data on plant community composition, growth, survival, and so forth are available before expanding wolf populations settle in new areas. The sentinel approach enabled us to bypass these site legacy effects by randomizing our site selection and using the same plant species at all locations (Quirion and Blossey 2023). This allowed us to capture the effect that unbrowsed oaks and goldenrod showed higher growth at sites with increased wolf density (Figure 4). This improved growth suggests differences in resource availability among our sites, with wolves preferentially settling and occupying nutrient‐rich sites, most likely in response to higher prey, that is, deer abundance. Finally, we randomized our site selection across the entirety of northern Wisconsin and sampled 42 sites, far exceeding the number of sites included in previous studies, and we measured wolf effects across a continuous gradient of wolf density and residence time, rather than a binary (e.g., wolf vs. no wolf) or categorical (e.g., high, medium, low wolf use) classification. Continuous correlations between wolf density, herbivory rates, and plant growth provide stronger evidence of a top‐down effect, if it exists, than a categorical representation of predators (Ford and Goheen 2015), in part because there is more information and power and a decreased risk of type I error.

Although we acknowledge the “chicken and egg” problem applies to our plant community data, the results demonstrating a decline in the frequency of 11 common herbaceous understory species as wolf density increased (Figure 6) are further evidence for the lack of a developing or existing trophic cascade in northern Wisconsin. We further caution that, even if a trophic cascade were to develop, we should not expect rapid change in plant community composition in forest understories. Many of the characteristic northern hardwood understory plants are characterized by long lifespans, low reproductive rates, and long juvenile periods (Jolls and Whigham 2014). Demographic responses to land‐use change or reduction in deer browse pressure will take decades, if not longer, to be measurable as a change in plant community composition (Flinn and Vellend 2005; Kahmen and Jules 2005; Griffiths and McGee 2018). In fact, traditional vegetation assessments typically fail to capture change at the plant community level, even when deer are completely excluded by fencing for over a decade (Habeck and Schultz 2015; Nuzzo et al. 2017).

Our work, the statistical analyses we conducted, and our interpretation of the results reflect several key challenges in ecology, particularly our ability to tease apart drivers of change in ecosystem processes, species abundance, and communities without the availability of experimental controls. Primary producers in northern Wisconsin exist in a working landscape dominated by forests, but with a legacy of clear‐cutting, farming, farm abandonment, increasing deer populations, and predator eradication and return, all of which continue to affect plant individuals. Climate change, non‐native species, forestry operations, and recreational activities further impact plant populations, requiring individuals to respond simultaneously to various stressors to enable their local persistence (Tylianakis et al. 2008; Tscharntke et al. 2012). Teasing out the strength of a potential wolf effect, given the multitude of factors influencing the presence and abundance of primary producers, herbivores, and their predators, is a prime example of the challenges in ecological research.

**FIGURE 7:**
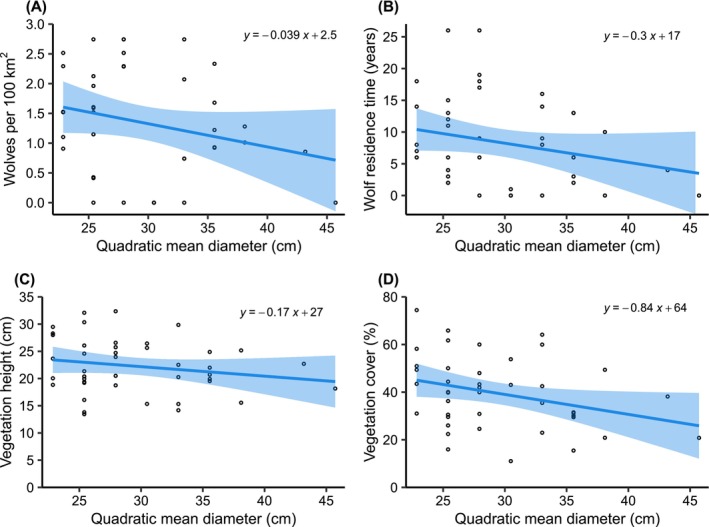
Plots of (A) wolf density, (B) wolf residence time, (C) vegetation height, and (D) vegetation cover as they relate to tree size (quadratic mean diameter). Solid lines and shaded 95% confidence intervals are from simple linear models of the response as a function of tree size. The linear equation is included for each plot.

Although we developed our approach to evaluate whether wolves would be able to trigger a trophic cascade and assist in managing deer populations, we discovered other factors that were predictors of deer browse probability and matched the strength of the effects of wolf density or wolf residence time (Figure 2). Browse probability was highest at sites with the largest tree sizes (high QMD, Figure 4C), which reflects an older forest age structure (e.g., 80–100‐year‐old trees). These sites also had the lowest wolf density and wolf residence time (Figure 7), but also had shorter understory plants and less vegetative cover, and were associated with introduced earthworm presence (Table 10, Figure 7). Introduced earthworms are facilitated by deer, increase plant growth, elevate plant nutrient levels, benefit some plants but not others, and are involved in various interactions that we are only slowly beginning to understand (Dávalos, Simpson, et al. 2015; Frelich et al. 2019; Dobson et al. 2024). Reduced vegetative cover, as found in these older forests, may indicate lower resource availability or a deer cropping effect, and likely made our planted seedlings more visible to foraging deer, although we did find an interactive effect with wolf density for goldenrod (Figure 4). In contrast, sites with younger trees (lower QMD, representing 40–50‐year‐old trees) were either in transition to becoming forests or had been harvested when the first wolves dispersed to northern Wisconsin about 60 years ago. These young trees and other herbaceous plants offered prime deer habitat with abundant food and cover. Thus, they were also a perfect place for wolves because of high prey availability. It is also possible that these sites have higher soil nutrient availability and higher productivity, allowing prolonged agricultural use before being abandoned (Larson and Larson 2016; Yu and Lu 2018). That unbrowsed oaks and goldenrods grow better at these sites supports our reasoning of higher resource availability (Table 7), but we lack historic and detailed land‐use information for our sites.

**TABLE 10:** Test statistics of Pearson's correlations comparing tree size (quadratic mean diameter) to habitat characteristics of study sites in northern Wisconsin.

Variable	*r*	*t*‐value	DF	*p*‐value
Wolf density	−0.24	−1.6	40	0.12
Wolf residence time	−0.24	−1.6	40	0.12
Vegetation height	−0.20	−1.3	40	0.21
Vegetation cover	−0.32	−2.1	40	0.04
Summer precipitation	0.09	0.6	40	0.56
Leaf litter depth	−0.08	−0.5	40	0.61
Earthworm presence	0.12	0.8	40	0.45

Our own work, and that of many others, has advanced our understanding of ecological networks and assembly rules that shape current and future plant communities in northern hardwood forests, but also documented the enormous complexities of ecological interactions (Bellemare et al. 2002; Foster and Aber 2004; Ellison et al. 2005). Deer are one of the main drivers of change in understory plant communities in the region (Wiegmann and Waller 2006; Frerker et al. 2013). Reversing the negative effects of high deer populations will require deer reductions, and the idea of a predator‐triggered trophic cascade is appealing to scientists, foresters, and wildlife managers. Unfortunately, our data suggest that the effect of wolves on deer abundance and/or foraging behavior, even in a largely forested landscape, is not powerful enough to quickly reverse the negative impacts of chronic browsing in hardwood forests and protect biodiversity long‐term. There is a glimmer of hope in our data that suggests that wolves may have the potential to enhance the protection of palatable herbaceous and woody plants in the presence of high vegetative cover (Figure 4F). Managers seeking to restore predators to an ecosystem should be aware that any cascading effects may be too weak to reverse negative herbivore impacts unless parallel management efforts are taken. Human interventions will be required to achieve improved ecological outcomes. However, with the projections of hunter numbers, managers are faced with the challenge of finding novel methods of intervention that are socially acceptable and financially feasible.

## Author Contributions

**Elaine M. Brice:** conceptualization (lead), data curation (lead), formal analysis (lead), project administration (lead), visualization (lead), writing – original draft (lead), writing – review and editing (lead). **Jennifer Price Tack:** conceptualization (supporting), data curation (supporting), writing – review and editing (supporting). **Amanda M. McGraw:** conceptualization (supporting), data curation (supporting), resources (supporting), writing – review and editing (supporting). **Bernd Blossey:** conceptualization (lead), data curation (lead), funding acquisition (lead), methodology (equal), project administration (lead), supervision (lead), writing – original draft (lead), writing – review and editing (equal).

## Conflicts of Interest

The authors declare no conflicts of interest.

## Data Availability

The data that support the findings of this study are openly available in Dryad at https://doi.org/10.5061/dryad.905qfttwv.

## Appendix A

**TABLE A1:** Habitat types classified within the Mesic group by the Wisconsin Department of Natural Resources. The first part of the name, before the “/” represents the dominant tree species in the potential climax association, and the second part is the characteristic understory. Both AVb‐V and ACl are considered dry mesic sites and were chosen while in the field after the originally selected neighboring mesic site was unusable.

Code	Name	Growth potential	Number of sites
AFVb	*Acer‐Fagus/Viburnum*	Good	1
ATM	*Acer‐Tsuga/Maianthemum*	Good	16
ATFD	*Acer‐Tsuga‐Fagus/Dryopteris*	Good	0
ATFSt	*Acer‐Tsuga‐Fagus/Streptopus*	Good	0
AAs	*Acer/Arisaema*	Excellent	2
ATD	*Acer‐Tsuga/Dryopteris*	Excellent	5
ATDH	*Acer‐Tsuga/Dryopteris‐Hydrophyllum*	Excellent	1
AHVb	*Acer/Hydrophyllum‐Viburnum*	Excellent	0
AFAd	*Acer‐Fagus/Adiantum*	Excellent	0
AFAl	*Acer‐Fagus/Allium*	Excellent	0
ACaCi	*Acer/Caulophylium‐Circaea*	Excellent	1
AOCa	*Acer/Osmorhiza‐Caulophyllum*	Excellent	12
AH	*Acer/Hydrophyllum*	Excellent	2
Avb‐V	*Acer/Viburnum‐Vaccinium variant*	Fair	1
ACl	*Acer/Clintonia*	Fair	1

**TABLE A2:** Variance inflation factors (VIF) for parameters in the best approximating models of oak and goldenrod browsing and growth.

Response	Parameter	VIF	
		Oak model	Goldenrod model
Browse	Species: *Q. rubra*	1.062	
	Planting height	1.063	1.027
	Veg. cover	1.025	1.078
	Earthworms	1.045	1.106
	Precipitation	1.018	1.026
	QMD	1.126	1.086
	Wolf residence time	1.096	
	Wolf density		1.130
	Veg. cover ‐x‐ Wolf density		1.075
Growth	Species: *Q. rubra*	1.161	
	Deer browse	1.079	1.106
	Other damage	1.035	
	Planting height	1.174	1.053
	Veg. cover	1.110	1.095
	Leaf litter	1.109	1.058
	Precipitation	1.110	1.049
	QMD	1.095	1.075
	Wolf density	1.313	1.392
	Browse ‐x‐ Wolf density	1.264	1.362

**TABLE A3:** Generalized variance inflation factors (GVIF) for parameters in the best approximating model of the plant community.

Parameter	GVIF	DF	GVIF^(1/(2*DF))^
Species	5.90	10	1.09
Veg. cover	1.33	1	1.15
Earthworms	1.46	1	1.21
Leaf litter	1.66	1	1.29
Precipitation	1.41	1	1.19
QMD	3.59	1	1.90
Deer browse	1.65	1	1.29
Wolf density	1.38	1	1.17
Species ‐x‐ QMD	18.67	10	1.16

## References

[ece372266-bib-0001] Allen, B. L. , L. R. Allen , H. Andrén , et al. 2017. “Can We Save Large Carnivores Without Losing Large Carnivore Science?” Food Webs 12: 64–75.

[ece372266-bib-0002] Allen, B. L. , P. J. S. Fleming , L. R. Allen , R. M. Engeman , G. Ballard , and L. K.‐P. Leung . 2013. “As Clear as Mud: A Critical Review of Evidence for the Ecological Roles of Australian Dingoes.” Biological Conservation 159: 158–174.

[ece372266-bib-0003] Alverson, W. S. , D. M. Waller , and S. L. Solheim . 1988. “Forests Too Deer: Edge Effects in Northern Wisconsin.” Conservation Biology 2: 348–358.

[ece372266-bib-0004] Averill, K. M. , D. A. Mortensen , E. A. H. Smithwick , et al. 2018. “A Regional Assessment of White‐Tailed Deer Effects on Plant Invasion.” AoB Plants 10: plx047. 29340133 10.1093/aobpla/plx047 PMC5761582

[ece372266-bib-0005] Averill, K. M. , D. A. Mortensen , E. A. H. Smithwick , and E. Post . 2016. “Deer Feeding Selectivity for Invasive Plants.” Biological Invasions 18: 1247–1263.

[ece372266-bib-0006] Bates, D. , M. Mächler , B. Bolker , and S. Walker . 2015. “Fitting Linear Mixed‐Effects Models Using lme4.” Journal of Statistical Software 67: 1–51.

[ece372266-bib-0007] Bee, J. N. , A. J. Tanentzap , W. G. Lee , et al. 2009. “The Benefits of Being in a Bad Neighbourhood: Plant Community Composition Influences Red Deer Foraging Decisions.” Oikos 118: 18–24.

[ece372266-bib-0008] Bellemare, J. , G. Motzkin , and D. R. Foster . 2002. “Legacies of the Agricultural Past in the Forested Present: An Assessment of Historical Land‐Use Effects on Rich Mesic Forests.” Journal of Biogeography 29: 1401–1420.

[ece372266-bib-0009] Bernes, C. , B. Macura , B. G. Jonsson , et al. 2018. “Manipulating Ungulate Herbivory in Temperate and Boreal Forests: Effects on Vegetation and Invertebrates. A Systematic Review.” Environmental Evidence 7: 13.

[ece372266-bib-0010] Berry, S. L. , L. A. Shipley , R. A. Long , and C. Loggers . 2019. “Differences in Dietary Niche and Foraging Behavior of Sympatric Mule and White‐Tailed Deer.” Ecosphere 10: e02815.

[ece372266-bib-0011] Blossey, B. , P. Curtis , J. Boulanger , and A. Dávalos . 2019. “Red Oak Seedlings as Indicators of Deer Browse Pressure: Gauging the Outcome of Different White‐Tailed Deer Management Approaches.” Ecology and Evolution 9: 13085–13103. 31871631 10.1002/ece3.5729 PMC6912884

[ece372266-bib-0012] Blossey, B. , A. Dávalos , and V. Nuzzo . 2017. “An Indicator Approach to Capture Impacts of White‐Tailed Deer and Other Ungulates in the Presence of Multiple Associated Stressors.” AoB Plants 9: plx034. 28894567 10.1093/aobpla/plx034 PMC5585856

[ece372266-bib-0013] Blossey, B. , and D. Hare . 2022. “Myths, wishful thinking, and accountability in predator conservation and management in the United States.” Frontiers in Conservation Science 3: 881483.

[ece372266-bib-0014] Blossey, B. , D. Hare , and D. M. Waller . 2024. “Where Have All the Flowers Gone? A Call for Federal Leadership in Deer Management in the United States.” Frontiers in Conservation Science 5: 1382132.

[ece372266-bib-0015] Bouchard, K. , J. E. Wiedenhoeft , A. P. Wydeven , and T. P. Rooney . 2013. “Wolves Facilitate the Recovery of Browse‐Sensitive Understory Herbs in Wisconsin Forests.” Boreal Environment Research 18: 7.

[ece372266-bib-0016] Bradshaw, L. , M. Pluemer , B. Beardmore , R. Holsman , and J. Petchenik . 2021. Hunters' Attitudes and Behaviors Related to Chronic Wasting Disease (CWD) in Wisconsin in 2019 Technical Report, Wisconsin Department of Natural Resources, Madison, WI.

[ece372266-bib-0017] Bradshaw, L. , and D. M. Waller . 2016. “Impacts of White‐Tailed Deer on Regional Patterns of Forest Tree Recruitment.” Forest Ecology and Management 375: 1–11.

[ece372266-bib-0018] Brice, E. M. , E. J. Larsen , and D. R. MacNulty . 2022. “Sampling Bias Exaggerates a Textbook Example of a Trophic Cascade.” Ecology Letters 25: 177–188. 34748261 10.1111/ele.13915 PMC9298920

[ece372266-bib-0019] Brice, E. M. , E. J. Larsen , D. R. Stahler , and D. R. MacNulty . 2024. “The Primacy of Density‐Mediated Indirect Effects in a Community of Wolves, Elk, and Aspen.” Ecological Monographs 95: e1627.

[ece372266-bib-0021] Callan, R. , N. P. Nibbelink , T. P. Rooney , J. E. Wiedenhoeft , and A. P. Wydeven . 2013. “Recolonizing Wolves Trigger a Trophic Cascade in Wisconsin (USA).” Journal of Ecology 101: 837–845.

[ece372266-bib-0022] Champagne, E. , J.‐P. Tremblay , and S. D. Côté . 2016. “Spatial Extent of Neighboring Plants Influences the Strength of Associational Effects on Mammal Herbivory.” Ecosphere 7: e01371.

[ece372266-bib-0023] Chips, M. J. , E. H. Yerger , A. Hervanek , et al. 2015. “The Indirect Impact of Long‐Term Overbrowsing on Insects in the Allegheny National Forest Region of Pennsylvania.” Northeastern Naturalist 22: 782–797.

[ece372266-bib-0024] Comeau, P. G. , and J. L. Heineman . 2003. “Predicting Understory Light Microclimate From Stand Parameters in Young Paper Birch ( *Betula papyrifera* Marsh.) Stands.” Forest Ecology and Management 180: 303–315.

[ece372266-bib-0025] Côté, S. D. , T. P. Rooney , J.‐P. Tremblay , C. Dussault , and D. M. Waller . 2004. “Ecological Impacts of Deer Overabundance.” Annual Review of Ecology, Evolution, and Systematics 35: 113–147.

[ece372266-bib-0026] Craine, J. M. , and R. Dybzinski . 2013. “Mechanisms of Plant Competition for Nutrients, Water and Light.” Functional Ecology 27: 833–840.

[ece372266-bib-0027] Craven, D. , M. P. Thakur , E. K. Cameron , et al. 2017. “The Unseen Invaders: Introduced Earthworms as Drivers of Change in Plant Communities in North American Forests (a Meta‐Analysis).” Global Change Biology 23: 1065–1074. 27590777 10.1111/gcb.13446 PMC5324548

[ece372266-bib-0028] Daigle, C. , M. Crête , L. Lesage , J.‐P. Ouellet , and J. Huot . 2004. “Summer Diet of Two White‐Tailed Deer, *Odocoileus virginianus* , Populations Living at Low and High Density in Southern Québec.” Canadian Field‐Naturalist 118: 360.

[ece372266-bib-0029] Dávalos, A. , V. Nuzzo , and B. Blossey . 2015. “Interactive Effects of Deer, Earthworms and Non‐Native Plants on Rare Forest Plant Recruitment.” Biological Conservation 187: 173–181.

[ece372266-bib-0030] Dávalos, A. , E. Simpson , V. Nuzzo , and B. Blossey . 2015. “Non‐Consumptive Effects of Native Deer on Introduced Earthworm Abundance.” Ecosystems 18: 1029–1042.

[ece372266-bib-0031] DelGiudice, G. D. , K. R. McCaffery , D. E. Beyer , and M. E. Nelson . 2009. “Prey of Wolves in the Great Lakes Region.” In Recovery of Gray Wolves in the Great Lakes Region of the United States, edited by A. P. Wydeven , T. R. Van Deelen , and E. J. Heske , 155–173. Springer New York.

[ece372266-bib-0032] Dobson, A. , and B. Blossey . 2015. “Earthworm Invasion, White‐Tailed Deer and Seedling Establishment in Deciduous Forests of North‐Eastern North America.” Journal of Ecology 103: 153–164.

[ece372266-bib-0033] Dobson, A. , A. Bowe , V. Nuzzo , A. Dávalos , T. Fahey , and B. Blossey . 2024. “Individual and Combined Effects of Non‐Native Earthworms and Native White‐Tailed Deer on Understorey Plant Survival, Growth and Reproduction.” Journal of Ecology 112: 1039–1057.

[ece372266-bib-0034] Dobson, A. , J. Richardson , and B. Blossey . 2020. “Effects of Earthworms and White‐Tailed Deer on Roots, Arbuscular Mycorrhizae, and Forest Seedling Performance.” Ecology 101: e02903. 31563154 10.1002/ecy.2903

[ece372266-bib-0035] Dobson, A. M. , B. Blossey , and J. B. Richardson . 2017. “Invasive Earthworms Change Nutrient Availability and Uptake by Forest Understory Plants.” Plant and Soil 421: 175–190.

[ece372266-bib-0036] Ellison, A. M. , M. S. Bank , B. D. Clinton , et al. 2005. “Loss of Foundation Species: Consequences for the Structure and Dynamics of Forested Ecosystems.” Frontiers in Ecology and the Environment 3: 479–486.

[ece372266-bib-0037] Estes, J. A. , J. Terborgh , J. S. Brashares , et al. 2011. “Trophic Downgrading of Planet Earth.” Science 333: 301–306. 21764740 10.1126/science.1205106

[ece372266-bib-0038] Fischer, S. , R. Johnson , G. Stauffer , et al. 2022. Wisconsin Gray Wolf Monitoring Report 15 April 2021 Through 14 April 2022, 29. Bureau of Wildlife Management, Wisconsin Department of Natural Resources.

[ece372266-bib-0039] Flagel, D. G. , G. E. Belovsky , and D. E. Beyer . 2016. “Natural and Experimental Tests of Trophic Cascades: Gray Wolves and White‐Tailed Deer in a Great Lakes Forest.” Oecologia 180: 1183–1194. 26670677 10.1007/s00442-015-3515-z

[ece372266-bib-0040] Fleming, P. J. S. 2019. “They Might be Right, but Beschta et al. (2018) Give No Strong Evidence that “Trophic Cascades Shape Recovery of Young Aspen in Yellowstone National Park”: A Fundamental Critique of Methods.” Forest Ecology and Management 454: 1–3.

[ece372266-bib-0041] Flinn, K. M. , and M. Vellend . 2005. “Recovery of Forest Plant Communities in Post‐Agricultural Landscapes.” Frontiers in Ecology and the Environment 3: 243–250.

[ece372266-bib-0042] Ford, A. T. , and J. R. Goheen . 2015. “Trophic Cascades by Large Carnivores: A Case for Strong Inference and Mechanism.” Trends in Ecology & Evolution 30: 725–735. 26498385 10.1016/j.tree.2015.09.012

[ece372266-bib-0043] Foster, D. R. , and J. D. Aber . 2004. Forests in time: The environmental consequences of 1,000 years of change in New England. Yale University Press.

[ece372266-bib-0126] Fox, J. , and S. Weisberg . 2019. An R Compantion to Applied Regression.

[ece372266-bib-0044] Frelich, L. E. , B. Blossey , E. K. Cameron , et al. 2019. “Side‐Swiped: Ecological Cascades Emanating From Earthworm Invasions.” Frontiers in Ecology and the Environment 17: 502–510. 31908623 10.1002/fee.2099 PMC6944502

[ece372266-bib-0045] Frerker, K. , A. Sabo , and D. Waller . 2014. “Long‐Term Regional Shifts in Plant Community Composition Are Largely Explained by Local Deer Impact Experiments.” PLoS One 9: e115843. 25551827 10.1371/journal.pone.0115843 PMC4281217

[ece372266-bib-0046] Frerker, K. , G. Sonnier , and D. M. Waller . 2013. “Browsing Rates and Ratios Provide Reliable Indices of Ungulate Impacts on Forest Plant Communities.” Forest Ecology and Management 291: 55–64.

[ece372266-bib-0047] Fuller, T. K. 1989. “Population Dynamics of Wolves in North‐Central Minnesota.” Wildlife Monographs 105: 3–41.

[ece372266-bib-0048] Gable, T. D. , S. K. Windels , J. G. Bruggink , and S. M. Barber‐Meyer . 2018. “Weekly Summer Diet of Gray Wolves ( *Canis lupus* ) in Northeastern Minnesota.” American Midland Naturalist 179: 15–27.

[ece372266-bib-0049] Geho, E. M. , D. Campbell , and P. A. Keddy . 2007. “Quantifying Ecological Filters: The Relative Impact of Herbivory, Neighbours, and Sediment on an Oligohaline Marsh.” Oikos 116: 1006–1016.

[ece372266-bib-0050] Gerhardt, F. , and D. R. Foster . 2002. “Physiographical and Historical Effects on Forest Vegetation in Central New England, USA.” Journal of Biogeography 29: 1421–1437.

[ece372266-bib-0051] Griffiths, G. R. , and G. G. McGee . 2018. “Lack of Herbaceous Layer Community Recovery in Postagricultural Forests Across Three Physiographic Regions of New York.” Journal of the Torrey Botanical Society 145: 1–20.

[ece372266-bib-0052] Guries, R. , J. Auer , S. Seaborne , et al. 2019. Strategic Plan 2019–2029: Wisconsin Forest Genetics Program. Wisconsin Department of Natural Resources.

[ece372266-bib-0053] Habeck, C. W. , and A. K. Schultz . 2015. “Community‐Level Impacts of White‐Tailed Deer on Understorey Plants in North American Forests: A Meta‐Analysis.” AoB Plants 7: plv119. 26487676 10.1093/aobpla/plv119 PMC4676796

[ece372266-bib-0054] Halaj, J. , and D. H. Wise . 2001. “Terrestrial Trophic Cascades: How Much Do They Trickle?” American Naturalist 157: 262–281. 10.1086/319190 18707289

[ece372266-bib-0055] Hartig, F. 2021. DHARMa: Residual Diagnostics for Hierarchical (Multi‐Level/Mixed) Regression Models.

[ece372266-bib-0056] Hobbs, N. T. , D. B. Johnston , K. N. Marshall , E. C. Wolf , and D. J. Cooper . 2024. “Does Restoring Apex Predators to Food Webs Restore Ecosystems? Large Carnivores in Yellowstone as a Model System.” Ecological Monographs 94: e1598.

[ece372266-bib-0057] Jenkins, L. H. , B. D. Murray , M. A. Jenkins , and C. R. Webster . 2015. “Woody Regeneration Response to Over a Decade of Deer Population Reductions in Indiana State Parks.” Journal of the Torrey Botanical Society 142: 205–219.

[ece372266-bib-0058] Jolls, C. L. , and D. F. Whigham . 2014. “Populations of and Threats to Tare Plants of the Herb Layer: Still More Challenges and Opportunities for Conservation Biologists.” In The Herbaceous Layer in Forests of Eastern North America, edited by F. S. Gilliam , 2nd ed., 134–200. Oxford University Press.

[ece372266-bib-0059] Kahmen, A. , and E. S. Jules . 2005. “Assessing the Recovery of a Long‐Lived Herb Following Logging: *Trillium ovatum* Across a 424‐Year Chronosequence.” Forest Ecology and Management 210: 107–116.

[ece372266-bib-0060] Karbstein, K. , C. Römermann , F. Hellwig , and K. Prinz . 2023. “Population Size Affected by Environmental Variability Impacts Genetics, Traits, and Plant Performance in *Trifolium montanum* L.” Ecology and Evolution 13: e10376. 37560178 10.1002/ece3.10376 PMC10406824

[ece372266-bib-0061] Kauffman, M. J. , J. F. Brodie , and E. S. Jules . 2010. “Are Wolves Saving Yellowstone's Aspen? A Landscape‐Level Test of a Behaviorally Mediated Trophic Cascade.” Ecology 91: 2742–2755. 20957967 10.1890/09-1949.1

[ece372266-bib-0062] Keddy, P. A. 1992. “Assembly and Response Rules: Two Goals for Predictive Community Ecology.” Journal of Vegetation Science 3: 157–164.

[ece372266-bib-0063] Kirkland, H. , D. Hare , M. Daniels , et al. 2021. “Successful Deer Management in Scotland Requires Less Conflict Not More.” Frontiers in Conservation Science 2: 770303.

[ece372266-bib-0064] Kohn, B. E. , and J. J. Mooty . 1971. “Summer Habitat of White‐Tailed Deer in North‐Central Minnesota.” Journal of Wildlife Management 35: 476–487.

[ece372266-bib-0065] Konôpka, B. , J. Pajtík , and L. A. Shipley . 2018. “Intensity of Red Deer Browsing on Young Rowans Differs Between Freshly‐Felled and Standing Individuals.” Forest Ecology and Management 429: 511–519.

[ece372266-bib-0066] Larson, L. E. , and B. A. Larson . 2016. The Enduring Cutover. Contributions to the History of Wisconsin's Northern Region, 440. Chequamegon Bay History.

[ece372266-bib-0067] Lenth, R. V. 2023. emmeans: Estimated Marginal Means.

[ece372266-bib-0068] Leopold, A. , L. K. Sowls , and D. L. Spencer . 1947. “A Survey of Over‐Populated Deer Ranges in the United States.” Journal of Wildlife Management 11: 162–177.

[ece372266-bib-0069] Massé, A. , and S. D. Côté . 2013. “Spatiotemporal Variations in Resources Affect Activity and Movement Patterns of White‐Tailed Deer (*Odocoileus virginianus*) at High Density.” Canadian Journal of Zoology 91: 252–263.

[ece372266-bib-0070] Maxwell, J. D. , A. C. Rhodes , and S. B. St. Clair . 2019. “Human Altered Disturbance Patterns and Forest Succession: Impacts of Competition and Ungulate Herbivory.” Oecologia 189: 1061–1070. 30887106 10.1007/s00442-019-04370-8

[ece372266-bib-0071] McCaffery, K. R. , J. Tranetzki , and J. Piechura . 1974. “Summer Foods of Deer in Northern Wisconsin.” Journal of Wildlife Management 38: 215–219.

[ece372266-bib-0072] McEwan, R. W. , J. M. Dyer , and N. Pederson . 2011. “Multiple Interacting Ecosystem Drivers: Toward an Encompassing Hypothesis of Oak Forest Dynamics Across Eastern North America.” Ecography 34: 244–256.

[ece372266-bib-0073] McNamara, S. , R. Johnson , J. Price‐Tack , et al. 2021. Wisconsin Gray Wolf Monitoring Report 15 April 2020 Through 14 April 2021, 28. Bureau of Wildlife Management, Wisconsin Department of Natural Resources.

[ece372266-bib-0074] Miller, K. M. , and B. J. McGill . 2019. “Compounding Human Stressors Cause Major Regeneration Debt in Over Half of Eastern US Forests.” Journal of Applied Ecology 56: 1355–1366.

[ece372266-bib-0075] Miller, K. M. , S. J. Perles , J. P. Schmit , et al. 2023. “Overabundant Deer and Invasive Plants Drive Widespread Regeneration Debt in Eastern United States National Parks.” Ecological Applications 33: e2837. 36890590 10.1002/eap.2837

[ece372266-bib-0076] Mladenoff, D. J. , M. K. Clayton , S. D. Pratt , T. A. Sickley , and A. P. Wydeven . 2009. “Change in Occupied Wolf Habitat in the Northern Great Lakes Region.” In Recovery of Gray Wolves in the Great Lakes Region of the United States, edited by A. P. Wydeven , T. R. Van Deelen , and E. J. Heske , 119–138. Springer New York.

[ece372266-bib-0077] Motta, R. 2003. “Ungulate Impact on Rowan ( *Sorbus aucuparia* L.) and Norway Spruce ( *Picea abies* (L.) *Karst*.) Height Structure in Mountain Forests in the Eastern Italian Alps.” Forest Ecology and Management 181: 139–150.

[ece372266-bib-0078] Muhly, T. B. , M. Hebblewhite , D. Paton , J. A. Pitt , M. S. Boyce , and M. Musiani . 2013. “Humans Strengthen Bottom‐Up Effects and Weaken Trophic Cascades in a Terrestrial Food Web.” PLoS One 8: e64311. 23667705 10.1371/journal.pone.0064311 PMC3648482

[ece372266-bib-0079] Nagy, C. , C. Ng , N. Veverka , and M. Weckel . 2022. “Assessment of a 15‐Year White‐Tailed Deer Management Program and Woody Recovery in a Suburban Forest Preserve.” Forest Ecology and Management 503: 119748.

[ece372266-bib-0080] Nielsen, C. K. , and J. K. Stroud‐Settles . 2018. “Home Range and Habitat Use of Female White‐Tailed Deer ( *Odocoileus virginianus* ) in the Northern Lower Peninsula of Michigan.” Mammal Study 43: 179–185.

[ece372266-bib-0081] Nilsen, E. B. , E. J. Milner‐Gulland , L. Schofield , A. Mysterud , N. C. Stenseth , and T. Coulson . 2007. “Wolf Reintroduction to Scotland: Public Attitudes and Consequences for Red Deer Management.” Proceedings of the Royal Society B: Biological Sciences 274: 995–1003. 10.1098/rspb.2006.0369 PMC2141678 17264063

[ece372266-bib-0082] Nogués‐Bravo, D. , D. Simberloff , C. Rahbek , and N. J. Sanders . 2016. “Rewilding Is the New Pandora's Box in Conservation.” Current Biology 26: R87–R91. 26859272 10.1016/j.cub.2015.12.044

[ece372266-bib-0083] Nuzzo, V. , A. Dávalos , and B. Blossey . 2017. “Assessing Plant Community Composition Fails to Capture Impacts of White‐Tailed Deer on Native and Invasive Plant Species.” AoB Plants 9: plx026. 28775829 10.1093/aobpla/plx026 PMC5534022

[ece372266-bib-0084] O'Neil, S. T. , D. E. Beyer , and J. K. Bump . 2019. “Territorial Landscapes: Incorporating Density‐Dependence Into Wolf Habitat Selection Studies.” Royal Society Open Science 6: 190282. 31827818 10.1098/rsos.190282 PMC6894554

[ece372266-bib-0085] Pace, M. L. , J. J. Cole , S. R. Carpenter , and J. F. Kitchell . 1999. “Trophic Cascades Revealed in Diverse Ecosystems.” Trends in Ecology & Evolution 14: 483–488. 10542455 10.1016/s0169-5347(99)01723-1

[ece372266-bib-0086] Paine, R. T. 1980. “Food Webs: Linkage, Interaction Strength and Community Infrastructure.” Journal of Animal Ecology 49: 667–685.

[ece372266-bib-0087] Painter, L. E. , R. L. Beschta , E. J. Larsen , and W. J. Ripple . 2018. “Aspen Recruitment in the Yellowstone Region Linked to Reduced Herbivory After Large Carnivore Restoration.” Ecosphere 9: e02376.

[ece372266-bib-0088] Patton, S. R. , M. B. Russell , M. A. Windmuller‐Campione , and L. E. Frelich . 2018. “Quantifying Impacts of White‐Tailed Deer ( *Odocoileus virginianus* Zimmerman) Browse Using Forest Inventory and Socio‐Environmental Datasets.” PLoS One 13: e0201334. 30138322 10.1371/journal.pone.0201334 PMC6107117

[ece372266-bib-0089] Pendergast, T. H. , S. M. Hanlon , Z. M. Long , A. A. Royo , and W. P. Carson . 2016. “The Legacy of Deer Overabundance: Long‐Term Delays in Herbaceous Understory Recovery.” Canadian Journal of Forest Research 46: 362–369.

[ece372266-bib-0090] Porter, W. F. , and H. B. Underwood . 1999. “Of Elephants and Blind Men: Deer Management in the U.S. National Parks.” Ecological Applications 9: 3–9.

[ece372266-bib-0091] Potvin, M. J. , T. D. Drummer , J. A. Vucetich , D. E. Beyer , R. O. Peterson , and J. H. Hammill . 2005. “Monitoring and Habitat Analysis for Wolves in Upper Michigan.” Journal of Wildlife Management 69: 1660–1669.

[ece372266-bib-0092] QGIS Development Team . 2023. QGIS Geographic Information System. QGIS Association.

[ece372266-bib-0093] Quirion, B. R. , and B. Blossey . 2023. “Experimental Evaluation of Four Protocols for Assessing White‐Tailed Deer Browse Intensity.” Ecological Indicators 154: 110651.

[ece372266-bib-0094] Ramirez, J. I. , P. A. Jansen , and L. Poorter . 2018. “Effects of Wild Ungulates on the Regeneration, Structure and Functioning of Temperate Forests: A Semi‐Quantitative Review.” Forest Ecology and Management 424: 406–419.

[ece372266-bib-0095] Reed, S. P. , D. R. Bronson , J. A. Forrester , et al. 2023. “Linked Disturbance in the Temperate Forest: Earthworms, Deer, and Canopy Gaps.” Ecology 104: e4040. 36960924 10.1002/ecy.4040

[ece372266-bib-0096] Renaud, P. C. , H. Verheyden‐Tixier , and B. Dumont . 2003. “Damage to Saplings by Red Deer ( *Cervus elaphus* ): Effect of Foliage Height and Structure.” Forest Ecology and Management 181: 31–37.

[ece372266-bib-0097] Ripple, W. J. , J. A. Estes , R. L. Beschta , et al. 2014. “Status and Ecological Effects of the World's Largest Carnivores.” Science 343: 1241484. 24408439 10.1126/science.1241484

[ece372266-bib-0098] Ripple, W. J. , T. M. Newsome , C. Wolf , et al. 2015. “Collapse of the World's Largest Herbivores.” Science Advances 1: e1400103. 26601172 10.1126/sciadv.1400103 PMC4640652

[ece372266-bib-0099] Roder, S. , F. Biollaz , S. Mettaz , et al. 2020. “Deer Density Drives Habitat Use of Establishing Wolves in the Western European Alps.” Journal of Applied Ecology 57: 995–1008.

[ece372266-bib-0100] Rogers, D. A. , T. P. Rooney , T. J. Hawbaker , V. C. Radeloff , and D. M. Waller . 2009. “Paying the Extinction Debt in Southern Wisconsin Forest Understories.” Conservation Biology 23: 1497–1506. 19508676 10.1111/j.1523-1739.2009.01256.x

[ece372266-bib-0101] Rogers, L. L. , J. J. Mooty , and D. Dawson . 1981. Foods of White‐Tailed Deer in the Upper Great Lakes Region: A Review. General Technical Report, 1–24. U.S. Dept. of Agriculture, Forest Service, North Central Forest Experiment Station.

[ece372266-bib-0102] Romme, W. H. , M. G. Turner , G. A. Tuskan , and R. A. Reed . 2005. “Establishment, Persistence, and Growth of Aspen ( *Populus tremuloides* ) Seedlings in Yellowstone National Park.” Ecology 86: 404–418.

[ece372266-bib-0103] Rooney, T. P. 2001. “Deer Impacts on Forest Ecosystems: A North American Perspective.” Forestry 74: 201–208.

[ece372266-bib-0104] Rounds, R. C. 1979. “Height and Species as Factors Determining Browsing of Shrubs by Wapiti.” Journal of Applied Ecology 16: 227.

[ece372266-bib-0105] Royo, A. A. , D. W. Kramer , K. V. Miller , N. P. Nibbelink , and S. L. Stout . 2017. “Spatio‐Temporal Variation in Foodscapes Modifies Deer Browsing Impact on Vegetation.” Landscape Ecology 32: 2281–2295.

[ece372266-bib-0106] Royo, A. A. , P. Raymond , C. C. Kern , et al. 2023. “Desired REgeneration Through Assisted Migration (DREAM): Implementing a Research Framework for Climate‐Adaptive Silviculture.” Forest Ecology and Management 546: 121298.

[ece372266-bib-0107] Rushing, C. S. , R. W. Rohrbaugh , C. J. Fiss , C. S. Rosenberry , A. D. Rodewald , and J. L. Larkin . 2020. “Long‐Term Variation in White‐Tailed Deer Abundance Shapes Landscape‐Scale Population Dynamics of Forest‐Breeding Birds.” Forest Ecology and Management 456: 117629.

[ece372266-bib-0108] Schmitz, O. J. , P. A. Hambäck , and A. P. Beckerman . 2000. “Trophic Cascades in Terrestrial Systems: A Review of the Effects of Carnivore Removals on Plants.” American Naturalist 155: 141–153. 10.1086/303311 10686157

[ece372266-bib-0109] Silvertown, J. 2004. “Plant Coexistence and the Niche.” Trends in Ecology & Evolution 19: 605–611.

[ece372266-bib-0110] Stauffer, G. E. , N. M. Roberts , D. M. Macfarland , and T. R. Van Deelen . 2021. “Scaling Occupancy Estimates up to Abundance for Wolves.” Journal of Wildlife Management 85: 1410–1422.

[ece372266-bib-0111] Telford, S. R. 2017. “Deer Reduction Is a Cornerstone of Integrated Deer Tick Management.” Journal of Integrated Pest Management 8: 25.

[ece372266-bib-0112] Thornton, M. M. , R. Shrestha , Y. Wei , P. E. Thornton , S. Kao , and B. E. Wilson . 2020. Daymet: Daily Surface Weather Data on a 1‐Km Grid for North America. ORNL DAAC.

[ece372266-bib-0113] Tscharntke, T. , J. M. Tylianakis , T. A. Rand , et al. 2012. “Landscape Moderation of Biodiversity Patterns and Processes – Eight Hypotheses.” Biological Reviews 87: 661–685. 22272640 10.1111/j.1469-185X.2011.00216.x

[ece372266-bib-0114] Tylianakis, J. M. , R. K. Didham , J. Bascompte , and D. A. Wardle . 2008. “Global Change and Species Interactions in Terrestrial Ecosystems.” Ecology Letters 11: 1351–1363. 19062363 10.1111/j.1461-0248.2008.01250.x

[ece372266-bib-0115] U.S. Fish and Wildlife Service, and U.S. Census Bureau . 2016. National Survey of Fishing, Hunting, and Wildlife‐Associated Recreation, 144. U.S. Department of the Interior, Fish and Wildlife Service.

[ece372266-bib-0116] Wardle, D. A. , G. M. Barker , G. W. Yeates , K. I. Bonner , and A. Ghani . 2001. “Introduced Browsing Mammals in New Zealand Natural Forests: Aboveground and Belowground Consequences.” Ecological Monographs 71: 587–614.

[ece372266-bib-0117] WDNR . 2001. Chapter 4: Forest Legacy Program. Wisconsin Department of Natural Resources.

[ece372266-bib-0118] WDNR . 2013. “Chapter 40: Northern Hardwood Cover Type.” In Page Silviculture Handbook, 2431.5. Wisconsin Department of Natural Resources.

[ece372266-bib-0119] Wiegmann, S. M. , and D. M. Waller . 2006. “Fifty Years of Change in Northern Upland Forest Understories: Identity and Traits of “Winner” and “Loser” Plant Species.” Biological Conservation 129: 109–123.

[ece372266-bib-0120] Wilson, C. J. 2004. “Could We Live With Reintroduced Large Carnivores in the UK?” Mammal Review 34: 211–232.

[ece372266-bib-0121] Winnie, J. A. 2012. “Predation Risk, Elk, and Aspen: Tests of a Behaviorally Mediated Trophic Cascade in the Greater Yellowstone Ecosystem.” Ecology 93: 2600–2614. 23431591 10.1890/11-1990.1

[ece372266-bib-0122] Wojcik, B. , S. Lee , G. Freeman , and D. J. Storm . 2021. White‐Tailed Deer Population Status 2021, 8. Wisconsin Department of Natural Resources.

[ece372266-bib-0123] Wolf, C. , and W. J. Ripple . 2018. “Rewilding the World's Large Carnivores.” Royal Society Open Science 5: 172235. 29657815 10.1098/rsos.172235 PMC5882739

[ece372266-bib-0124] Wydeven, A. P. , J. E. Wiedenhoeft , R. N. Schultz , et al. 2009. “History, Population Growth, and Management of Wolves in Wisconsin.” In Recovery of Gray Wolves in the Great Lakes Region of the United States, edited by A. P. Wydeven , T. R. Van Deelen , and E. J. Heske , 87–105. Springer New York.

[ece372266-bib-0125] Yu, Z. , and C. Lu . 2018. “Historical Cropland Expansion and Abandonment in the Continental U.S. During 1850 to 2016.” Global Ecology and Biogeography 27: 322–333.

